# Iridium Complexes with BIAN-Type Ligands: Synthesis, Structure and Redox Chemistry

**DOI:** 10.3390/ijms241310457

**Published:** 2023-06-21

**Authors:** Nikolai F. Romashev, Ivan V. Bakaev, Veronika I. Komlyagina, Pavel A. Abramov, Irina V. Mirzaeva, Vladimir A. Nadolinny, Alexander N. Lavrov, Nikolai B. Kompan’kov, Artem A. Mikhailov, Iakov S. Fomenko, Alexander S. Novikov, Maxim N. Sokolov, Artem L. Gushchin

**Affiliations:** 1 Nikolaev Institute of Inorganic Chemistry SB RAS, Novosibirsk 630090, Russia; nikolaj.romashev75@gmail.com (N.F.R.); i.bakaev@g.nsu.ru (I.V.B.); v.komlyagina@g.nsu.ru (V.I.K.); abramov@niic.nsc.ru (P.A.A.); dairdre@gmail.com (I.V.M.); spectr@niic.nsc.ru (V.A.N.); lavrov@niic.nsc.ru (A.N.L.); nmr124@niic.nsc.ru (N.B.K.); fomenko@niic.nsc.ru (I.S.F.); caesar@niic.nsc.ru (M.N.S.); 2 Department of Natural Sciences, Novosibirsk State University, Novosibirsk 630090, Russia; 3 Research School of Chemistry and Applied Biomedical Sciences, Tomsk Polytechnic University, Tomsk 634034, Russia; 4 Laboratoire de Cristallographie, Résonance Magnétique et Modélisations, Université de Lorraine, CNRS, CRM2, UMR 7036, 54000 Nancy, France; amikhailov@niic.nsc.ru; 5 Institute of Chemistry, Saint Petersburg State University, Saint Petersburg 199034, Russia; 6 Research Institute of Chemistry, Peoples’ Friendship University of Russia (RUDN University), Moscow 117198, Russia

**Keywords:** iridium, BIAN, iridium(II), nitrosyl complexes, synthesis, cyclic voltammetry, redox-active ligands, non-innocence, crystal structure, EPR spectroscopy, static magnetic susceptibility, redox isomerism, DFT calculations

## Abstract

A series of iridium complexes with bis(diisopropylphenyl)iminoacenaphtene (dpp-bian) ligands, [Ir(cod)(dpp-bian)Cl] (**1**), [Ir(cod)(NO)(dpp-bian)](BF_4_)_2_ (**2**) and [Ir(cod)(dpp-bian)](BF_4_) (**3**), were prepared and characterized by spectroscopic techniques, elemental analysis, X-ray diffraction analysis and cyclic voltammetry (CV). The structures of **1**–**3** feature a square planar backbone consisting of two C = C π-bonds of 1,5-cyclooctadiene (cod) and two nitrogen atoms of dpp-bian supplemented with a chloride ion (for **1**) or a NO group (for **2**) to complete a square-pyramidal geometry. In the nitrosyl complex **2**, the Ir-N-O group has a bent geometry (the angle is 125°). The CV data for **1** and **3** show two reversible waves between 0 and -1.6 V (vs. Ag/AgCl). Reversible oxidation was also found at E_1/2_ = 0.60 V for **1**. Magnetochemical measurements for **2** in a range from 1.77 to 300 K revealed an increase in the magnetic moment with increasing temperature up to 1.2 μ_B_ (at 300 K). Nitrosyl complex **2** is unstable in solution and loses its NO group to yield [Ir(cod)(dpp-bian)](BF_4_) (**3**). A paramagnetic complex, [Ir(cod)(dpp-bian)](BF_4_)_2_ (**4**), was also detected in the solution of **2** as a result of its decomposition. The EPR spectrum of **4** in CH_2_Cl_2_ is described by the spin Hamiltonian Ĥ = gβHŜ with S = 1/2 and g_xx_ = g_yy_ = 2.393 and g_zz_ = 1.88, which are characteristic of the low-spin 5d^7^-Ir(II) state. DFT calculations were carried out in order to rationalize the experimental results.

## 1. Introduction

Bis(imino)-acenaphthenes (BIANs) belong to a well-studied class of aromatic acceptor diimines that exhibit extraordinarily rich coordination and redox chemistries [1,2,3,4]. They contain a central 1.4-diazabutadiene fragment supplemented with a naphthalene backbone. This combination leads to a combination of strong σ-donor and π-acceptor properties, which ensures the stabilization of metal ions in both high and low oxidation states. In addition, the aromatic naphthalene fragment forces the anti–anti conformation upon the α-diimine fragment, thereby promoting strong chelation with a metal center. BIANs form complexes with almost all main group elements [5,6,7,8,9,10] and transition metals [11,12,13,14,15,16,17,18,19,20]. A key feature of BIANs is their ability to accept up to four electrons and/or reversibly exchange electrons with the coordinating metal, which can trigger various redox transformations. Redox isomerism or valence tautomerism was found for some BIAN complexes [21,22]. Unsurprisingly, metal complexes with BIANs catalyze a large number of organic transformations. Among these are olefin polymerization reactions as well as reduction reactions: hydrogenation [15,23,24,25,26,27,28], reduction of nitroarenes [29,30,31] and hydroamination [32,33,34]. There are also several examples of metal/BIAN-catalyzed oxidations [31,35,36,37].

Despite the impressive number of published results, the coordination properties of BIANs across the periodic table have been studied extremely unevenly. Most attention has been paid to complexes of the main group elements and transition metals of the 3d series. At the same time, complexes of 4d and 5d elements with BIANs have been studied rather selectively. A large number of works on palladium/BIAN complexes stands out against the background of a small number of works on the chemistry of group 9 elements—rhodium and iridium—for which the number of structurally characterized coordination compounds is no more than a dozen. Only recently, we reported mono- and binuclear complexes of rhodium(I) and rhodium(III) with 1,2-bis[(2,6-diisopropylphenyl)-imino]acenaphthene (dpp-bian): [Rh(cod)(dpp-bian)Cl], *mer*-[Rh(dpp-bian)(H_2_O)Cl_3_] and [Rh_2_(dpp-bian)_2_(µ-Cl)_2_] [38,39,40]. The *mer*-[Rh(dpp-bian)(H_2_O)Cl_3_] complex catalyzes the electrochemical reduction of CO_2_ [39]. Several Ir(III) complexes with BIANs are known. Among them are cyclopentadienyl complexes of the type [ƞ^5^-Cp*Ir(BIAN)Cl]^+^ (BIAN = mes-bian [41], Ph-bian [42] and ClPh-bian [43]), which catalyze the hydrogenation of terephthalaldehyde and the hydroamination of 2-(2-phenylethynyl)aniline and heteroleptic [(ppy)_2_Ir(BIAN)]^+^ complexes [44,45]. Complexes of Ir(I) with BIAN ligands have not been reported. Meanwhile, the combination of a redox-active BIAN and an iridium ion as a late transition metal can lead to non-additive electronic properties of the resulting complex due to the energy proximity of the d-orbitals of the metal and the frontier orbitals of the redox-active ligand. In this case, uncertainty can be expected in assigning the oxidation states both to the metal and to the ligand, and BIANs can be considered non-innocent ligands. In particular, this work aimed at stabilizing the low oxidation states of Ir (I and even II) with sterically bulky dpp-bian and studying its non-innocent properties.

## 2. Results and Discussion

**Figure 1:**
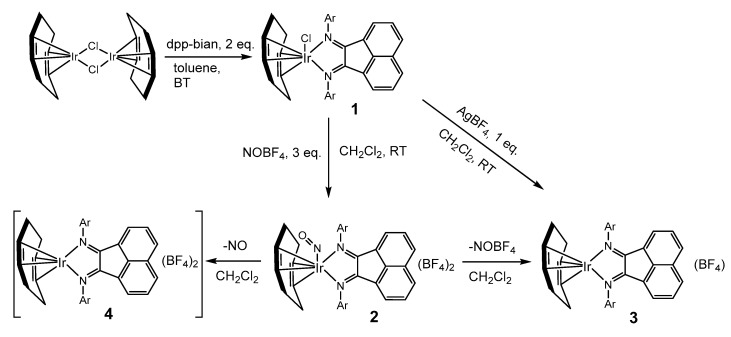
Synthetic routes toward complexes **1**–**4**.

***Synthesis and characterization.*** The general scheme for the synthesis of complexes **1–3** is shown in Figure 1. The interaction of the dinuclear complex [Ir_2_(cod)_2_(µ-Cl)_2_] with dpp-bian in a 1:2 molar ratio under mild conditions leads to cleavage of the Ir-Cl bridges with the formation of a mononuclear complex [Ir(cod)(dpp-bian)Cl] (**1**) and a 76% yield. Treatment of **1** with NOBF_4_ (3 eq.) gave the nitrosyl complex [Ir(cod)(NO)(dpp-bian)](BF_4_)_2_ (**2**) with a 66% yield. Complex **2** is unstable in CH_2_Cl_2_ and decomposes to yield [Ir(cod)(dpp-bian)](BF_4_) (**3**). This was confirmed by UV-vis spectroscopy data (Figure S1). Complex **3** was obtained with a 90% yield by a direct reaction between **1** and AgBF_4_ taken in a 1:1 molar ratio. The analytical purity of **1–3** was confirmed by CHN microanalysis. Complexes **1–3** were characterized by FT-IR and ^1^H NMR spectroscopy. The structures of **1–3** were determined by X-ray diffraction analysis. Paramagnetic complex **4** was also detected in a solution of **2** using EPR spectroscopy (see below). It was not isolated as an individual compound. The instability of **2** toward the loss of the NO group hindered its further characterization.

^1^H NMR spectra of complexes **1** and **3** (Figures S2 and S3) show characteristic signals from the isopropyl groups of dpp-bian at 0.85, 1.32 and 3.77 ppm (for **1**) and 0.97, 1.52 and 3.44 ppm (for **3**). The signals of the CH_3_ groups of isopropyl groups are wide for **1** because their mobility is hindered by interaction with the Cl ligand. This is not observed for a similar Rh complex [38], which dissociates in solution with the elimination of Cl^-^, or for **3**, for which the corresponding signals are narrow and with clear doublet structures. The signals from dpp-bian aromatic protons were in the range of 6.5–8.5 ppm. Characteristic signals from coordinated cyclooctadiene were found in the region of 1.92–4.24 ppm.

The FT-IR spectrum of **1** displays the characteristic stretching vibrations ν(C=N) at 1549 cm^−1^ and ν(C-C) at 1497 cm^−1^ of the dpp-bian ligand (Figure S4). The position of these absorption bands agrees with both the neutral and the radical anion oxidation state of dpp-bian. On the contrary, ν(C = N) and ν(C-C) stretching vibrations for **2** and **3** were found in the ranges of 1575–1672 cm^−1^ and 1469–1472 cm^−1^, which unequivocally correspond to the neutral state of dpp-bian (Figures S5 and S6). The band of NO stretching in **2** appeared at 1721 cm^−1^, which is typical for nitrosyl complexes with bent NO groups [46,47]. In the FT-IR spectra of **2** and **3**, a broad strong absorption band in the region of 1000–1200 cm^−1^ was attributed to the vibrations of the BF_4_^-^ anion.

### 2.1. X-ray Structure Description

**Figure 2:**
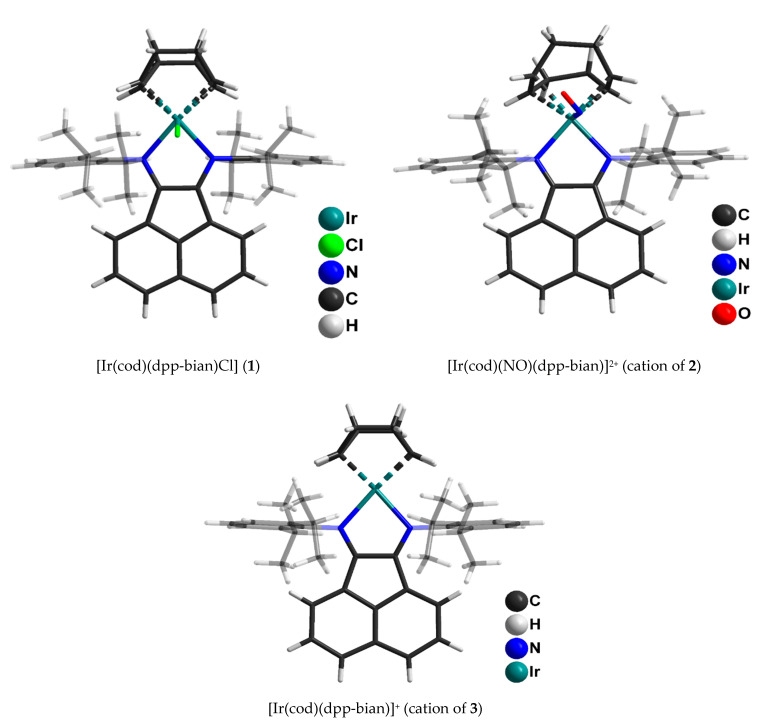
Molecular structures of (**1**), cation of (**2**) and cation of (**3**) determined by X-ray diffraction analysis.

Single crystals of **1** suitable for X-ray diffraction analysis were obtained by slow evaporation from a dichloromethane/toluene mixture. Single crystals of **2** and **3** suitable for X-ray diffraction analysis were obtained from dichloromethane/hexane (**2**) and dichloromethane/ether (**3**) solvent mixtures. The molecular structures of **1**–**3** are shown in Figure 2 and Figure S7. The main geometric parameters are summarized in Table S2.

Complex **1** has a distorted square-pyramidal structure of the coordination node. The coordination environment of iridium consists of two dpp-bian nitrogen atoms, midpoints of two cyclooctadiene π-bonds and an axial chlorine atom. Noteworthy is the somewhat elongated Ir-Cl bond (2.480 Å), which, however, is shorter (by 0.1 Å) than the one in the rhodium analogue, [Rh(cod)(dpp-bian)Cl] (Rh-Cl, 2.580 Å) [38]. In the coordinated dpp-bian, the C-C bonds (1.458 Å) are somewhat shortened and the C = N bonds (1.314 Å) are elongated when compared with the bonds in [Rh(cod)(dpp-bian)Cl] (C-C, 1.486 Å and C = N, 1.294 Å), which might indicate a more delocalized nature of the electron density in the diimine fragment. The Ir-N bond lengths are 2.080 Å, which falls within the range of Ir-N bond lengths for similar complexes [48,49,50].

Complex **2** has a similar structure; the nitrogen atom of the NO group is located at the top of the square pyramid instead of the chlorine atom. The outer coordination sphere contains two BF_4_^-^ anions. The C-C and C = N bond lengths in the diimine moiety (1.50 Å and 1.30 Å, respectively) indicate the neutral state of dpp-bian. The Ir-N (dpp-bian) bond lengths are 2.12 Å, which is slightly longer than in complex **1**. The Ir-N (NO) distance is 1.95 Å, which falls within the range of the Ir-N bond lengths observed in iridium nitrosyl complexes [46,47]. The Ir-N-O angle is 125°, and the N-O bond length is 1.2 Å, which is typical for iridium nitrosyl complexes with a bent nitrosyl group [46,51,52].

The iridium coordination environment in complex **3** has a square geometry of two dpp-bian nitrogen atoms and midpoints of two cyclooctadiene π-bonds. The C-C and C = N bond lengths in dpp-bian (1.493 Å and 1.298 Å, respectively) correspond to the neutral state of the ligand. The Ir-N bond lengths are 2.095 Å, intermediate between those of **1** and **2**.

Crystal packing features intermolecular π–π interactions between acenaphthene fragments in **2** and **3**. This leads to the formation of dimers between the positively charged complex cations (Figures S8 and S9). These dimers form a crystal packing (Figures S10 and S11), in which anions and solvent molecules fill the free space. In the crystal packing in **2** and **3**, three pseudochannels oriented in the [001] direction of the crystal can be observed. Such dimers are absent in the crystal structure of **1**, probably due to the neutral charge of the complex. To theoretically study the intermolecular π–π interactions between acenaphthene fragments in the crystal structures **2** and **3**, DFT calculations followed by the topological analysis of the electron density distribution were carried out at the ωB97XD/DZP-DKH level of theory for model supramolecular associates (see the computational details and Table S13). The topological analysis of the electron density distribution in model supramolecular associates via the QTAIM approach revealed the presence of bond critical points for various intermolecular C···C contacts in the crystal structures **2** and **3** (Table S14). The low magnitude of the electron density (0.003–0.006 a.u.), the positive values of the Laplacian of electron density (0.009–0.018 a.u.) and the zero, or very close to zero, positive energy density (0.000–0.001 a.u.) in bond critical points for these intermolecular C···C contacts and their estimated strengths (0.3–0.6 kcal/mol) are typical for π-π and related interactions in similar chemical systems [53,54,55,56,57,58,59,60]. The balance between the kinetic energy density, G, and the potential energy density, V, at the bond critical points for intermolecular C···C contacts in the crystal structures **2** and **3** (viz., –G/V>1) reveals that these interactions are purely non-covalent [61], and negative values of λ_2_ confirm the attractive nature of these contacts [62,63]. The contour line diagrams of the Laplacian of electron density distribution, bond paths, and selected zero-flux surfaces; the visualization of the electron localization function (ELF); and the reduced density gradient (RDG) analyses for selected intermolecular C–C contacts in the crystal structures **2** and **3** are illustrated in Figures S12 and S13.

### 2.2. Redox Properties

**Figure 3:**
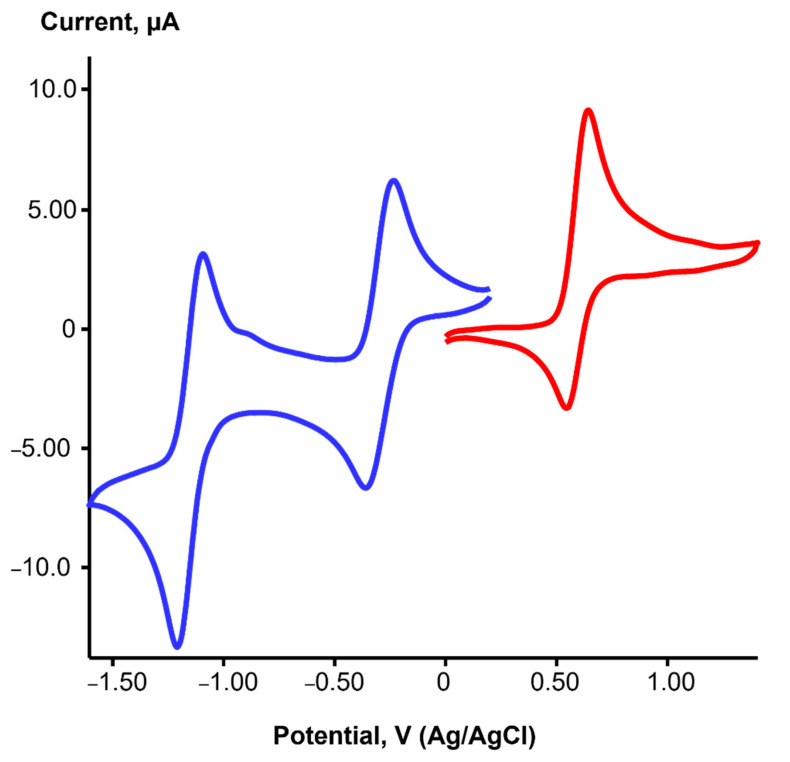
CV of **1** in CH_2_Cl_2_ in the −1.6–1.4 V region at a potential scan rate of 100 mV/s (blue spectrum—reduction part, red spectrum—oxidation part).

The redox properties of iridium complexes **1** and **3** were studied in CH_2_Cl_2_ using cyclic voltammetry (CV). The cyclic voltammogram of solution **1** (Figure 3) showed two quasi-reversible reduction waves at E_1/2_ = −0.30 V (ΔE = 110 mV) and E_1/2_ = −1.15 V (ΔE = 110 mV), as well as one quasi-reversible oxidation process at E_1/2_ = 0.60 V (ΔE = 96 mV). Likewise, two characteristic reversible reduction waves were detected in the CVs of rhodium(I), palladium(II) or platinum(II) complexes with dpp-bian [17,38]. These processes are considered ligand-centered and correspond to the sequential two-electron reduction of the BIAN ligand with the formation of the BIAN monoanion and the BIAN dianion. The results of the DFT calculations for **1** (see below) confirm the main contribution of dpp-bian (64%) to the lowest unoccupied MOs, although the contribution of Ir orbitals (23%) to LUMO is also significant.

On the contrary, the oxidation process could involve a metal-centered Ir^I^/Ir^II^ redox couple. However, the HOMO for **1** consists of only 42% of the Ir orbitals, with a significant contribution from the dpp-bian (34%). Therefore, this redox process can better be referred to as mixed-metal/ligand-centered oxidation. It is noteworthy that the oxidation process is reversible (quasi-reversible), which indicates a certain stability of the oxidation product.

Complex **3** has a similar reduction pattern (Figure S14) with two quasi-reversible reduction waves at E_1/2_ = −0.20 V (ΔE = 83 mV) and E_1/2_ = −1.14 V (ΔE = 70 mV). In addition, an irreversible oxidation process at E_a_ = 1.55 V was detected, with a significant anodic shift compared to **1**. The corresponding re-reduction peak was centered at E_c_ = 1.16 V. According to DFT calculations, the HOMO and LUMO for **3** are completely localized on the Ir (98%) and dpp-bian (99%), respectively. Therefore, the oxidation of **3** can be considered an exclusively metal-centered process which is irreversible, in contrast to **1**. Apparently, the involvement of the BIAN ligand in the oxidation process (as for **1**) is a key factor in ensuring its reversibility. Irreversible metal-centered oxidation was reported for the rhodium analog [Rh(cod)(dpp-bian)]^+^ [38]. Strong anodic shift (complex **3** is more difficult to oxidize than **1**) is consistent with the lower energy of the HOMO for **3**. The one-electron oxidation product of **3** should thus be an Ir(II) complex, [Ir^II^(cod)(dpp-bian)](BF_4_)_2_ (**4**), the formation of which has been proven by EPR (see below).

The anodic and cathodic peak potentials of the redox processes for **1** and **3** were almost independent of the potential scan rate (50–200 mV/s), which indicates an electrochemically reversible process. Moreover, the ratio between the peak current and the square root of the scan rate, I·ν^–1/2^ vs. scan rate, was constant, which is characteristic of a diffusion-controlled electron transfer reaction (Figures S15–S23 and Tables S15–S17).

### 2.3. Non-Innocent Properties of Dpp-Bian and NO Ligands in 1 and 2

As noted in the discussion of the structures **1–3**, the C-C and C = N distances within the dpp-bian ligand in **1** are not typical of the neutral state of dpp-bian. These values are better suited for the radical anion state of dpp-bian rather than the neutral one, unlike what was found for **2**, **3** and [Rh(cod)(dpp-bian)Cl] [38]. The positions of the ν(C = N) and ν(C-C) vibration bands in the FT-IR spectrum of **1** are consistent with the excess electron density on the dpp-bian. The formation of the dpp-bian radical anion could be explained by the electron density transfer from iridium(I) to the ligand. In this case, complex **1** should be considered as paramagnetic [Ir^II^(cod)(dpp-bian^•/-^)Cl] with two unpaired electrons, one on the iridium and one on the dpp-bian. However, magnetic susceptibility measurements have shown diamagnetic behavior of **1** down to 20 K, below which a weak paramagnetic contribution of iron impurities (~0.1 at. %) shows up. This absence of a significant paramagnetic response points to the singlet state of [Ir^I^(cod)(dpp-bian^0^)Cl] (*S* = 0). DFT calculations performed for **1** exclude a transition from the ground singlet state to the excited paramagnetic state (see the computational studies below) because the diamagnetic state was found to be 81 kJ/mol more favorable than the excited paramagnetic state. However, the calculated charge on the Ir atom was somewhat higher than that on the Rh atom in [Rh(cod)(dpp-bian)Cl] [38], and there was a small negative charge on the dpp-bian which could indicate a high degree of electron density delocalization between the metal and dpp-bian ligand in **1**. Based on these results, we believe that the charge state of the dpp-bian in complex **1** is best described as neutral in formal terms, although the outflow of the electron density on the redox-active ligand affects the C = N and C-C distances and the corresponding vibrational frequencies.

Reversible oxidation of **1** at moderate potential (see CV data) encouraged us to attempt the preparation of a paramagnetic Ir(II) complex [Ir^II^(cod)(dpp-bian)Cl]^+^ by one-electron oxidation of **1** with a suitable oxidizing agent—NOBF_4_, in this case. However, the reaction of **1** with NOBF_4_ resulted in the formation of a nitrosyl complex, **2**. At first glance, this reaction can be described as a non-isocharged substitution of NO^+^ for Cl^-^ with the preservation of the oxidation state of Ir(I) and the formation of [Ir^I^(cod)(NO^+^)(dpp-bian)](BF_4_)_2_. Most of the known iridium nitrosyl complexes are described as containing an NO^+^ group. On the other hand, we can also assume the oxidative addition of NO^+^ to Ir(I) with the formation of [Ir^III^(cod)(NO^-^)(dpp-bian)](BF_4_)_2_. There is an assumption that a bent NO configuration (the Ir-N-O angle is 125° in **2**) indicates a negative charge on NO [46,47,51,64]. For example, a square-pyramidal complex of iridium(III) [Ir(NO)(SH)_2_(PPh_3_)_2_] with a bent nitrosyl ligand was reported [51]. However, one must be aware of the limitations of such an approach. The M-NO bond is mainly covalent, and, depending on the total electronic count of the {M-NO} unit, the spin localization and the number of π-bonds in frontier orbitals, the formal charges of the metal and NO can vary significantly without change in the M-N-O angle [65,66,67]. The calculated Ir-N-O angle and other geometric parameters for 2 are in good agreement with the X-ray diffraction data. Thus, the ground state of complex 2 can be formally interpreted either as diamagnetic (singlet) [Ir^I^(cod)(NO^+^)(dpp-bian)](BF_4_)_2_ or [Ir^III^(cod)(NO^-^)(dpp-bian)](BF_4_)_2_.

Magnetochemical measurements in the static regime performed for **2** (see the magnetic details below) showed an increase in the magnetic moment with increasing temperature up to 1.2 μ_B_ (at 300 K), indicating the existence of a paramagnetic state. Thus, in addition to the ground singlet state for **2**, one might expect an excited triplet state. This could be an intramolecular charge-transfer transition (redox isomeric transition), given the presence of three centers in **2** that can change their valence state (Ir, NO and dpp-bian), or simply a singlet–triplet transition within one structural fragment of **2** without significant charge redistribution. One of the possible options for redox isomeric transition is the electron transfer from the NO group to Ir(III) with the formation of [Ir^II^(cod)(NO^0^)(dpp-bian)](BF_4_)_2_ with two paramagnetic centers: a d^7^-Ir(II) ion and a NO^0^ group. On the other hand, the singlet–triplet intraligand transition option looks more realistic, since the dpp-bian makes the main contribution to the frontier molecular orbitals of [Ir(cod)(NO)(dpp-bian)]^2+^ (the cation of **2**), although the calculated energy difference between the singlet and triplet states (113.0 kJ/mol) is even higher than in **1** (see below).

A paramagnetic species was also detected in the solution of 2 at 77 K by EPR spectroscopy (see the EPR spectroscopy details below). However, this ESR active species was a completely different complex from S = ½ and must be a decomposition product. Considering the instability of the nitrosyl complex 2 toward the loss of the NO group, we suggest two possible ways in which 2 might decompose (Figure 1). The first way involves the elimination of NO+ (as NOBF4) from 2 and the formation of a diamagnetic complex of Ir(I), [Ir^I^(cod)(dpp-bian)](BF_4_) (**3**), which was isolated and structurally characterized. Another possibility is the elimination of NO gas to form a paramagnetic complex [Ir^II^(cod)(dpp-bian)](BF_4_)_2_ (**4**) with S = ½, which is the ESR active species in the solution. In fact, the cracking of single crystals of 2 accompanied by the release of a gas (which must have been NO) was visually observed. The calculated Ir-NO bond dissociation energy was 173–179 kJ/mol, being due either to the elimination of the NO+ or the release of NO. This is much less than the calculated Ir-Cl bond dissociation energy (447 kJ/mol), which confirms the easiness of the elimination of the NO group. In addition, the calculated ESR parameters for 4 are in satisfactory agreement with the experimental data (see below). Thus, the EPR spectroscopy data can be associated with the decomposition of 2 in solution with the formation of the Ir(II) complex [Ir^II^(cod)(dpp-bian)](BF_4_)_2_ (4). Our attempts to isolate this paramagnetic complex as an individual phase were, however, unsuccessful, apparently because of its low stability. This indirectly agrees with the CV data, which indicated the irreversible oxidation of 3 and, consequently, the instability of 4 as a product of one-electron oxidation of 3.

### 2.4. EPR Spectroscopy

**Figure 4:**
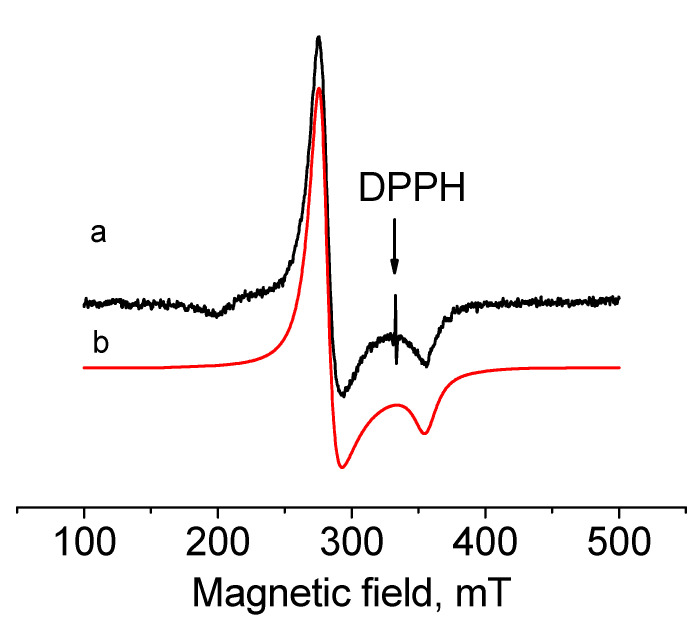
EPR spectra of **4** in CH_2_Cl_2_ recorded in the x-band at 77 K (a—experimental, b—simulated).

To detect the paramagnetic state of Ir(II) in [Ir^II^(cod)(dpp-bian)](BF_4_)_2_ (**4**), an EPR spectrum was recorded for a solution of **2** (the precursor of **4**) in CH_2_Cl_2_ at 77 K (Figure 4). The observed spectrum was well described by the spin Hamiltonian Ĥ = gβHŜ with S = 1/2 and the parameters g_xx_ = g_yy_ = 2.393 and g_zz_ = 1.88, which are characteristic of Ir(II) ion with the 5d^7^ electronic configuration. Close g-factor values were observed for the similar mononuclear Ir(II) complexes described by Fuchigami [68]. These are mixed-ligand complexes of Ir(II) with polypyridine and cod ligands, which have the following constants: g_x_ = 2.456, g_y_ = 2.346 and g_z_ = 1.933 for [(^Me^N4)Ir^II^(cod)]^2+^ and g_x_ = 2.604, g_y_ = 2.429 and g_z_ = 1.911 for [(^t-Bu^N4)Ir^II^(cod)]^2+^. In contrast to these and other mononuclear Ir(II) complexes [69,70], no hyperfine structure from nitrogen atoms was observed in the EPR spectrum of **4**. This is consistent with the DFT data, which showed almost zero spin charge on the nitrogen atoms (see below).

### 2.5. Magnetic Measurements

**Figure 5:**
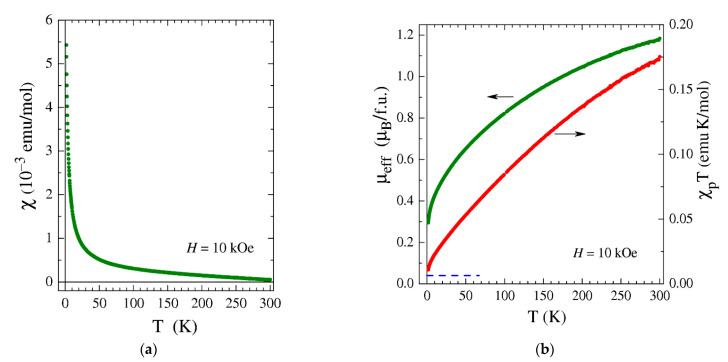
(**a**) Temperature dependence of the magnetic susceptibility, *χ*, measured for **2** at the magnetic field *H* = 10 kOe. (**b**) Temperature dependences of the effective moment, *μ*_eff_ (green symbols), and *χ*_p_*T* (red symbols) for **2.** The depicted *μ*_eff_ was calculated given an assumption of non-interacting magnetic moments (*θ* = 0). The estimated additive contribution of paramagnetic impurities to *χ*_p_*T* is indicated by the blue dashed line.

In contrast to the diamagnetic behavior of **1**, complex **2** turned out to be paramagnetic in the temperature range of 1.77–300 K (Figure 5a). Thermal cycles performed under both zero-field-cooling (ZFC) and field-cooling (FC) conditions demonstrated the magnetization of **2** to be perfectly reversible and reproducible, indicating the absence of any static magnetic order as well as the lack of detectable chemical degradation under experimental conditions.

Despite the simple appearance of *χ*(*T*), its interpretation is challenging. The paramagnetic component of the magnetic susceptibility, *χ*_p_(*T*), obtained by subtracting the diamagnetic contribution strongly deviates from the simple Curie–Weiss dependence, $\chi_{p}\left(T\right)=N_{A}\mu_{eff}^{2}/3k_{B}\left(T-\theta \right)$ (*N*_A_ and *k*_B_ are the Avogadro number and the Boltzmann constant), implying that the effective magnetic moment *µ*_eff_ is temperature-dependent. The calculated *µ*_eff_ decreases from ≈1.2 μ_B_ at *T* = 300 to ≈0.3 μ_B_ at *T* = 1.77 K (Figure 5b); the product *χ*_p_*T* behaves accordingly. Taking into account the high likelihood of paramagnetic iron impurities (from initial reagents, similar to the case of **1**) which provide an additive contribution to *χ*_p_*T* (the dashed line in Figure 5b), the low-temperature ground state of **2** can be considered nearly non-magnetic. Since the crystal structure of **2** cannot provide a considerable antiferromagnetic (AF) exchange interaction between the complex ions, these ions themselves should possess a non-magnetic, low-temperature ground state. The latter, however, is hard to reconcile with the high-temperature *χ*_p_(*T*) behavior. Indeed, the complexes of Ir(I) and Ir(III) with even numbers of electrons in the 5d shell (d^8^ and d^6^) have long been known to be exclusively diamagnetic owing to the strong crystal-field splitting that causes the spin-paired (*S* = 0) state of the respective Ir ions [71,72,73,74]. What one would expect for Ir(I) and Ir(III) complexes, in addition to the core diamagnetism, is just a weak Van Vleck paramagnetic contribution that should be strictly temperature-independent due to a large gap Δ>>*k*_B_*T* separating triplet levels from the occupied ground singlet one. In Ir(I) and Ir(III) complexes, the diamagnetic contribution usually overcomes the weak Van Vleck one, ensuring overall diamagnetic response; the complex **1** described above serves as such an example. The magnetic susceptibility of **2**, however, behaves differently: it is rather high at high temperatures and changes with *T* (Figure 5a), despite the fact that both the diamagnetic and Van Vleck terms should be *T*-independent. The observed *χ*_p_(*T*) behavior of **2** does not fit that of the Ir(II) and Ir(IV) complexes either. From the magnetic point of view, Ir(II) and Ir(IV) resemble ions with one unpaired electron (S = ½) [75]; their effective magnetic moment, *µ*_eff_, is known to exceed the spin-only value of 1.73 μ_B_ due to the spin–orbit coupling and to remain large even at low temperatures if the crystal structure ensures their magnetically dilute state [75,76].

The apparent controversy regarding the low-*T* and high-*T* magnetic behavior could be resolved by assuming either (i) a thermally assisted charge redistribution between the different structural fragments (Ir, NO and dpp-bian) of **2** or (ii) a thermally assisted singlet–triplet transition within one structural fragment of **2** without significant charge redistribution, which provides uncompensated magnetic moments at high temperatures. The former phenomenon is well known as redox isomerism or valence tautomerism and often occurs when a metal of variable valence is bound to a redox-active (non-innocent) ligand [77]. Cobalt complexes with o-dioxolene ligands and other redox-active ligands often display thermally or photo-induced valence tautomeric transitions [78,79,80,81]. In contrast, regarding their rhodium [82,83,84] and iridium [85] analogs, valence tautomerism is limited to a few examples. A recent paper [82] reported thermally induced electron transfer in a rhodium(I) dioxolene complex resulting in a mixed-valence Rh(I,II)-semiquinonato/catecholato state. A valence tautomeric equilibrium of [Rh^III^(LSPhISQ^•/-^)]/[Rh^II^(LSPhIQ)] was found in a cis-[Rh^III^(LSPhISQ^•/-^)(PPh_3_)Cl_2_] (LSPhISQ^•/-^ = 2,4-di-tert-butyl-N-[2-(phenylthio)]phenyl-o-imino-benzosemiquinonate anion radical; LSPhIQ = ando-iminobenzo-quinone) [83]. Regarding iridium, the only publication mentions the existence of two redox isomers for Ir(I) complexes of the type [(cod)Ir(AsEt_3_)(o-semiquinone)] [85].

Given that the magnetic moment observed at room temperature is much lower than expected for two unpaired electrons, only partial charge redistribution or population of the triplet state is achieved at that temperature. To confirm or refute the assumption of a thermally induced redox isomeric process, variable-temperature FT-IR and diffuse reflectance spectroscopic studies were applied. No significant changes in temperature were detected for either spectrum (Figures S24 and S25), which indicates the absence of charge redistribution and redox isomeric transition. In addition, DFT calculations (see below) predicted too large an energy gap between the singlet and triplet levels for the cation of **2**, which excludes the singlet–triplet transition. Thus, the nature of the magnetic behavior of **2** remains a mystery for now.

### 2.6. Computational Studies

To adequately describe the electronic structure of new iridium complexes and interpret their non-innocent behavior and spectral properties, DFT calculations were carried out. Atomic coordinates taken from experimental X-ray diffraction data were used as a starting point for geometry optimization of complexes **1**–**3**. All optimized structures had no imaginary vibrational frequencies, which indicates that they correspond to local minima on potential energy surfaces. The resulting optimized geometric parameters were in good agreement with the experimental structural data (Tables S3, S5 and S6). Selected data are shown in Table 1.

**Figure 6:**
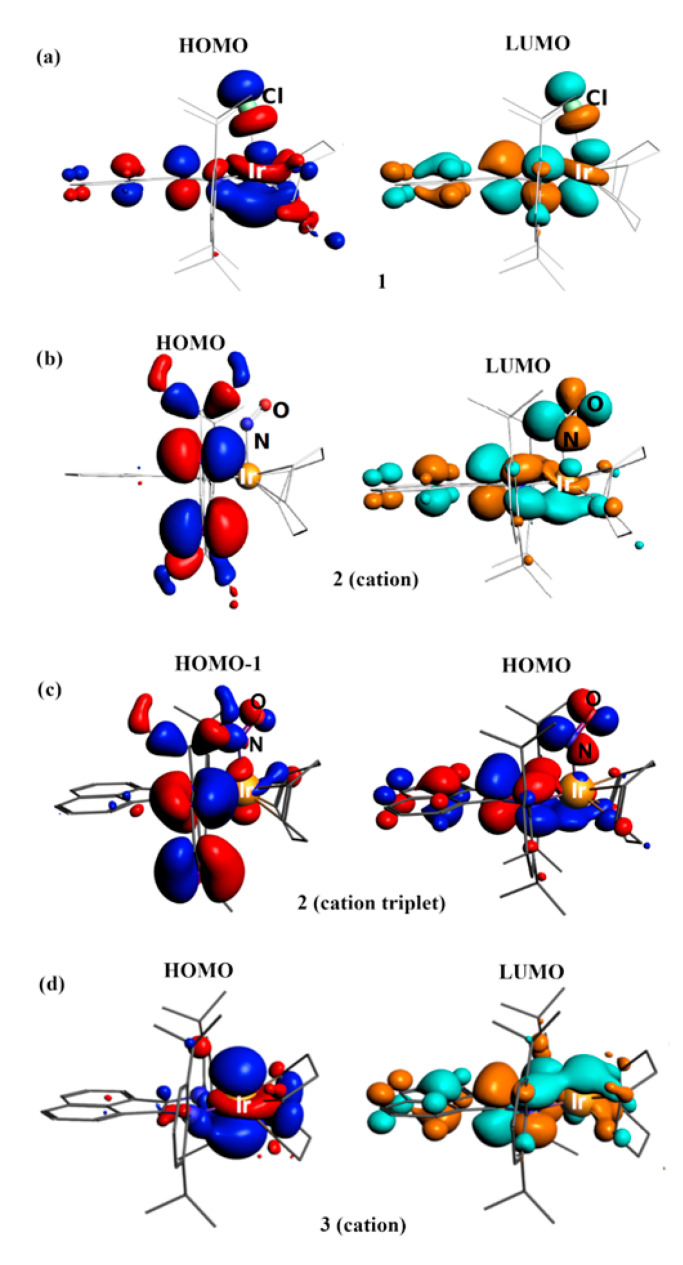
View of selected orbitals: (**a**) HOMO and LUMO for **1**, (**b**) HOMO and LUMO for cation [Ir(cod)(NO)(dpp-bian)]^2+^ of **2**, (**c**) HOMO-1 and HOMO for triplet state of cation [Ir(cod)(NO)(dpp-bian)]^2+^ of **2**, and (**d**) HOMO and LUMO for cation [Ir(cod)(dpp-bian)]^+^ of **3**.

The HOMO and LUMO of **1** are delocalized (Figure 6). However, the HOMO is more concentrated on Ir (42%), while the LUMO is mainly centered on the dpp-bian ligand (64%; Table 2). The HOMO-LUMO gap is 0.763 eV. A hypothetical excited paramagnetic state for **1** was also optimized, which turned out to be 80.8 kJ/mol less favorable than the singlet state. The calculated charges of the Ir atom were +0.85 (NBO) and +0.89 (AIM) (Table S4). These values are higher than for the Rh atom in the analogous [Rh(cod)(dpp-bian)Cl] complex (+ 0.71 and + 0.77). The NBO and AIM charge values for the dpp-bian ligand were −0.25 and −0.19, respectively. These values, as well as the delocalized nature of the frontier orbitals, indicate a partial transfer of electron density from Ir(I) to dpp-bian. This is consistent with the non-innocent behavior of the redox-active BIAN ligand.

Based on topological electron density analysis (Table S7), all Ir-ligand bonds in **1** are of an intermediate type (neither ionic nor covalent). The Ir-cod bonds are more covalent (higher potential-energy-density-to-kinetic-energy ratios for bond critical points) than both Ir-bian and Ir-Cl bonds. The anti-bonding nature of the HOMO with respect to the Ir-Cl interaction is responsible for the elongation of the Ir-Cl bond. However, this bond is still stronger than the Rh-Cl bond in [Rh(cod)(dpp-bian)Cl] [38] (Table S8).

**Figure 7:**
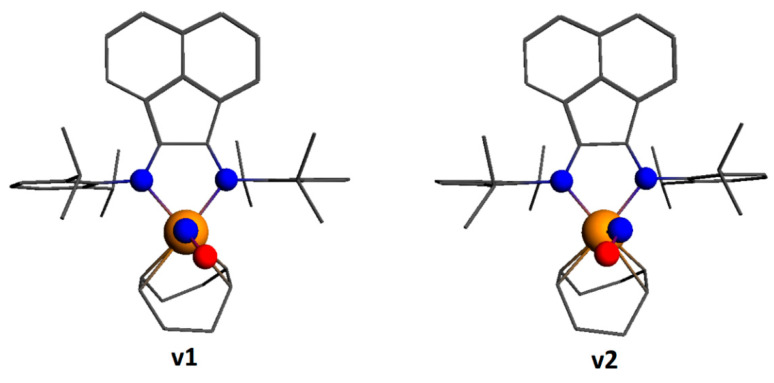
Two isomers of [Ir(cod)(NO)(dpp-bian)]^2+^ (cation of **2**).

The data of the X-ray diffraction analysis showed that the NO ligand in **2** was disordered; therefore, two possible isomers of [Ir(cod)(NO)(dpp-bian)]^2+^ (**v1** and **v2** in Figure 7) were optimized. They differed in the location of the NO group relative to the cod ligand. The **v1** isomer was only 3 kJ/mol more stable than the **v2** isomer. The ground state of both isomers is singlet. The corresponding excited paramagnetic states were also optimized. Since the difference between **v1** and **v2** was negligible, only the **v1** isomer was chosen for further consideration. The energy difference between the singlet and triplet states was 113.0 kJ/mol. The optimized Ir-N-O angle was 127.3°.

The LUMO of [Ir(cod)(NO)(dpp-bian)]^2+^ (the cation of **2**) is delocalized (Figure 6) but more concentrated on dpp-bian, as in 1, while the HOMO is completely localized on the aryl rings of the dpp-bian ligand. Thus, the HOMO can be considered as non-bonding Ir-L orbital. LUMO, LUMO+1 and LUMO+2 have significant NO ligand contributions (Table 2), especially LUMO+1. The HOMO–LUMO gap is 1.156 eV.

**Figure 8:**
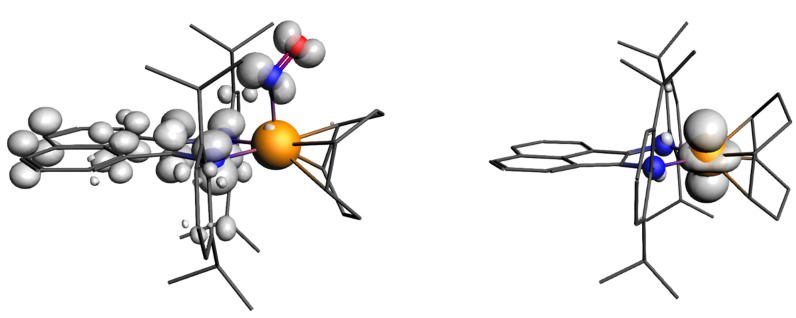
Spin density for paramagnetic **v1** isomer of [Ir(cod)(NO)(dpp-bian)]^2+^ (cation of **2**) and [Ir(cod)(dpp-bian)]^2+^ (cation of **4**).

Comparison of the NBO and AIM charges on the atoms (Table S9) for the ground and excited states of the cation of **2** showed that there was no significant charge transfer. The AIM charge on Ir became slightly more positive for **v1**, changing from 0.91 to 0.93, while the AIM charge on the NO group turned slightly more negative, changing from −0.06 to −0.09. The NBO charges showed the same trend. The charge on Ir was also only slightly greater than that of **1** (0.89; Table S4). Such small changes may not be interpreted as a change in the formal charges of the fragments. Notably, the spin density for the paramagnetic state of the cation of **2** is focused mainly on the dpp-bian π-system and the NO ligand (Figure 8). Based on these data, it is impossible to assign any reasonable formal charges to each fragment of the ground and excited states of the cation of **2**. However, it is well known that DFT calculations often do not allow the unambiguous determination of the formal oxidation states of metal atoms and NO ligands [71].

Since the instability of complex **2** due to the loss of the NO group was found experimentally, the Ir-NO bond dissociation energy was calculated. Two decomposition pathways for the cation of **2** were taken into account: the elimination of NO^+^ and the formation of [Ir(cod)(dpp-bian)]^+^ (the cation of **3**) and the elimination of NO^0^ and the formation of [Ir(cod)(dpp-bian)]^2+^ (the cation of **4**). The calculated difference between the formation energy of [Ir(cod)(NO)(dpp-bian)]^2+^ and the sum of the formation energies for NO^+^ and [Ir(cod)(dpp-bian)]^+^ was 178.93 kJ/mol. The difference between the formation energy of [Ir(cod)(NO)(dpp-bian)]^2+^ and the sum of the formation energies of NO^0^ and [Ir(cod)(dpp-bian)]^2+^ was 174.31 kJ/mol. The calculated Gibbs free energy difference was 140.45 kJ/mol for the first process, while it was 121.72 kJ/mol for the second one. These values are much lower than the energy required for the heterolytic breaking of the Ir-Cl bond (447 kJ/mol). Additionally, note that the transformation of **1** into **2** proceeds with the breaking of the Ir-Cl bond.

To confirm that the experimental EPR spectrum was indeed related to [Ir^II^(cod)(dpp-bian)]^2+^ (the cation of **4**) with S = ½, we performed calculations using the gauge-including atomic orbitals (GIAO) method [86], including both the scalar relativistic approach with perturbative spin–orbital effects and the two-component spin–orbit relativistic approach. The latter gave results closer to the experimental values (Table 3). The spin density resides almost entirely on Ir (Figure 8), which is consistent with the absence of a hyperfine structure due to splitting at the nitrogen atoms in the experimental EPR spectrum. The fragment analysis and the molecular orbital scheme for the cation of **4** (Figure S26) shows that the 10 highest occupied MOs were paired (five pairs) and were referred to the dpp-bian ligand. The unpaired levels were much lower and were referred to molecular orbitals with high Ir contributions.

## 3. Materials and Methods

Materials. All commercially available reagents—[Ir_2_(cod)_2_(µ-Cl)_2_] (Sigma Aldrich, Munich, Germany; 97%) and NOBF_4_ (Sigma Aldrich, Munich, Germany; 97%)—were used as purchased. 1,2-bis[(2,6-diisopropylphenyl)imino]acenaphthene (dpp-bian) was prepared according to the published procedure [87]. All organic solvents (CH_2_Cl_2_, hexane, diethyl ether and toluene) were dried by standard methods before use.

### 3.1. Physical Measurements

Elemental C, H and N analysis was performed with a EuroEA3000 Eurovector analyzer. IR spectra were recorded in the 4000–300 cm^–1^ range with a Perkin-Elmer System 2000 FTIR spectrometer (KBr pellets). ^1^H NMR spectra (500 MHz) were acquired on a Bruker Avance-500 spectrometer with a 5 mm PABBO-PLUS probe at room temperature. The chemical shifts were given in parts per million (ppm) from tetramethylsilane. The cyclic voltammograms (CVs) were recorded with a 797 VA Computrace system (Metrohm, Switzerland). All measurements were performed with a conventional three-electrode configuration consisting of glassy carbon working and platinum auxiliary electrodes and an Ag/AgCl/KCl reference electrode. The solvent used in all experiments was CH_2_Cl_2_, which was deoxygenated before use. Tetra-n-butylammonium hexafluorophosphate (0.1 M solution) was used as a supporting electrolyte. The concentration of the complexes was approx. 1 mM. Ferrocene was used as an internal standard, and the Fc/Fc^+^ potential was 0.49 V. The EPR spectra were recorded on a Varian E-109 spectrometer in the X-band at 77 K. The object of the study was the attainment of a solution of an iridium complex in CH_2_Cl_2_, and the DPPH radical with g = 2.0036 was used as a reference for calculating g factors. The EPR spectrum was modeled using the WINEPR SimFonia program. The magnetic properties of polycrystalline samples were studied using a Quantum Design MPMS-XL SQUID magnetometer in the temperature range of 1.77–300 K at magnetic fields *H* = 0–10 kOe. To determine the paramagnetic component of the molar magnetic susceptibility *χ*_p_(*T*), the temperature-independent diamagnetic contribution, *χ*_d_, and the possible contribution of ferromagnetic microimpurities, χ_F_, were subtracted from the measured values of the total molar susceptibility, *χ* = *M/H*: *χ*_p_(*T,H*) = *χ*(*T,H*)–*χ*_d_–*χ*_F_(*T,H*). The value of *χ*_d_ was calculated according to the additive Pascal scheme, while the ferromagnetic contribution, *χ*_F_, if any, was evaluated from the field dependences, *M*(*H*).

### 3.2. X-ray Crystallography

The crystallographic data and refinement details for **1**–**3** are given in Table S1. The diffraction data were collected for **1** on a Bruker Apex Duo diffractometer with CuK_α_ radiation (λ = 1.54178) by performing φ and ω scans of narrow (0.5°) frames at 150 K. Absorption correction was carried out empirically using SADABS (SADABS-2008/1) [88].

The diffraction data were collected for **2** and **3** on a Bruker D8 Venture diffractometer with a CMOS PHOTON III detector and an IµS 3.0 source (Mo Kα radiation, λ = 0.71073 Å) at 150 K. The φ- and ω-scan techniques were employed. Absorption correction was applied by SADABS (Bruker Apex3 software suite: Apex3, SADABS-2016/2 and SAINT, version 2018.7–2; Bruker AXS Inc.: Madison, WI, USA 2017.). Structures were solved by SHELXT [89] and refined by full-matrix least-squares treatment against |F|^2^ in anisotropic approximation with SHELX 2014/7 [90] in the ShelXle program [91]. H-atoms were refined in the geometrically calculated positions. The main geometrical parameters are summarized in Table S2. In the case of **2**, only one position of CH_2_Cl_2_ and one position of BF_4_^-^ was refined clearly. Other electronic densities were removed using the SQUEEZE procedure [92] of the PLATON program set [93]. This gave 125e in 286 Å^3^ per formula unit, which can be proposed as 1BF_4_ and 2CH_2_Cl_2_ per formula. The composition of **2** was established based on elemental analysis.

The crystallographic data were deposited in the Cambridge Crystallographic Data Centre under the deposition codes CCDC 1030978, 2189440 and 2189441.

### 3.3. Computational Details

The DFT calculations with the geometry optimization procedure for model systems were performed in ADF2021 [94] with GGA S12g functional [95], which includes dispersion corrections [96], with the all-electron triple-ζ basis set of Slater type functions with a set of polarization functions (TZP/ADF) [97]. Scalar relativistic effects (and, in some cases, spin–orbit effects) were included via the ZORA approach [98,99]. Closed-shell systems were treated with the restricted DFT approach, while open-shell systems were treated with the unrestricted DFT approach. Natural population analysis and bonding analysis were performed with NBO 6.0 [100]. The topology of electron density was studied in terms of AIM theory [101]. EPR parameters were calculated using the gauge-including atomic orbitals (GIAO) approach [86]. The results of the DFT calculations performed with the geometry optimization procedure are shown in Tables S3–S12. The DFT calculations based on the experimental X-ray geometries of **2** and **3** were carried out using the dispersion-corrected hybrid functional ωB97XD [102] with the help of the Gaussian-09 [103] program package. The Douglas–Kroll–Hess 2^nd^-order scalar relativistic calculations requested relativistic core Hamiltonian were carried out using the DZP-DKH basis sets [104,105,106,107] for all atoms. The topological analysis of the electron density distribution for studies of intermolecular π-π interactions in the crystal structures of **2** and **3** was performed using the Multiwfn program (version 3.7) [108]. The Cartesian atomic coordinates for model supramolecular associates are presented in Table S13.

### 3.4. Synthesis of [Ir(cod)(dpp-bian)Cl] (1)

Complex **1** was prepared under argon using the Schlenk technique. Dpp-bian (149 mg, 0.298 mmol) was added to a suspension of [Ir_2_(cod)_2_(µ-Cl)_2_] (100 mg, 0.149 mmol) in 20 mL of toluene. The mixture was stirred under reflux conditions for 24 h. The resulting emerald solution was evaporated in vacuum; the residue was dissolved in 10 mL of a dichloromethane/toluene 3:1 v/v mixture. An emerald crystalline product was obtained by allowing the solution to evaporate freely in air to 2 mL. Yield: 190 mg (76%). Anal. Calc. for C_44_H_52_ClN_2_Ir: C, 63.17; H, 6.27; N, 3.35%. Found: C, 63.0; H, 6.22; N, 3.2%. ^1^H NMR (500 MHz, 298 K, CD_2_Cl_2_): δ 0.85 (m, 12H) 1.32 (d, 12H), 1.92 (m, 4H), 2.32 (m, 4H), 3.77 (sep, 4H), 4.04 (m, 4H), 6.54 (d, 2H), 7.22 (m, 2H), 7.34 (m, 4H), 7.46 (m, 2H), 7.96 (d, 2H) ppm. IR (KBr, ν, cm^−1^): 3443 (m), 3059 (w), 3009 (w), 2959 (s), 2886 (s), 2877 (s), 2833 (w), 1549 (s), 1495 (s), 1464 (m), 1437 (m), 1416 (s), 1385 (w), 1362 (w), 1323 (w), 1305 (s), 1244 (w), 1206 (w), 1186 (w), 1161 (w), 1086 (w), 1066 (w), 1044 (w), 1026 (w), 1010 (w), 953 (w), 937 (w), 908 (w), 854 (w), 827 (s), 802 (m), 775 (s), 763 (m), 700 (w), 642 (w), 548 (w), 492 (m).

### 3.5. Synthesis of [Ir(cod)(NO)(dpp-bian)](BF_4_)_2_ (2)

Complex **2** was synthesized in the dark under argon using the Schlenk technique. NOBF_4_ (20 mg, 0.172 mmol) was added to a solution of **1** (50 mg, 0.060 mmol) in 10 mL of dichloromethane. The solution was stirred at room temperature for 24 h. The resulting brown solution was evaporated to 5 mL, and hexane was layered on top of the solution. The resulting brown crystalline product was washed with hexane and dried in vacuum. Yield: 40 mg (66%). Anal. Calc. for C_44_H_52_N_3_OB_2_F_8_Ir: C, 52.60; H, 5.21; N, 4.18%. Found: C, 52.3; H, 5.05; N, 4.3%. IR (KBr, ν, cm^−1^): 3224 (s), 2964 (s), 2926 (s), 2868 (m), 1721 (m), 1672 (m), 1622 (m), 1597 (m), 1575 (m), 1463 (s), 1436 (s), 1419 (s), 1389 (m), 1366 (w), 1300 (w), 1000–1200 (vs, br), 804 (m), 646 (w), 519 (w), 474 (m).

### 3.6. Synthesis of [Ir(cod)(dpp-bian)](BF_4_) (3)

AgBF_4_ (23.4 mg, 0.120 mmol) was added to a solution of complex **1** (100 mg, 0.120 mmol) in 20 mL of dichloromethane. The reaction mixture was stirred at room temperature for 24 h. The solution was evaporated and recrystallized from a dichloromethane/diethyl ether mixture to give a brown crystalline product. Yield: 95 mg (90%). Anal. Calc. for C_44_H_52_N_2_BF_4_Ir: C, 59.51; H, 5.90; N, 3.15%. Found: C, 59.7; H, 5.98; N, 3.2%. ^1^H NMR (500 MHz, 298 K, CDCl_3_): δ 0.97 (d, 12H) 1.52 (d, 12H), 1.95 (m, 4H), 2.32 (m, 4H), 3.44 (sep, 4H), 4.12 (m, 4H), 6.54 (d, 2H), 7.32 (m, 2H), 7.38 (m, 2H), 7.49 (m, 4H), 8.57 (d, 2H) ppm.IR (KBr, ν, cm^−1^): 3435 (br), 3060 (s), 2962 (m), 2926 (m), 2874 (w), 1626 (w), 1600 (m), 1578 (w), 1489 (w), 1466 (m), 1437 (m), 1420 (w), 1366 (w), 1327 (w), 1304 (m), 1252 (w), 1223 (w), 1184 (w), 1161 (w), 1000–1100 (vs), 895 (m), 833 (m), 802 (m), 781 (m), 761 (m), 646 (w), 621 (w), 546 (w), 519(w), 476 (w).

## 4. Conclusions

In this work, a series of novel iridium complexes, [Ir(cod)(dpp-bian)Cl] (**1**), [Ir(cod)(NO)(dpp-bian)](BF_4_)_2_ (**2**) and [Ir(cod)(dpp-bian)](BF_4_) (**3**), with bulky redox-active dpp-bian ligands were obtained and structurally characterized. Complex **2** is a rare example of an IrNO complex with a bent nitrosyl ligand. Regarding complexes **1** and **2**, an ambiguity in the determination of the charge states of both the iridium and the ligands (dpp-bian, NO) was found, which was the result of the non-innocent behavior of the dpp-bian and NO ligands. Complexes **1** and **3** demonstrate a reversible two-step, two-electron reduction typical of metal/BIAN complexes. In addition, a reversible mixed-metal/ligand-centered oxidation was detected for **1**. The magnetic properties of **2** in a range from 1.77 to 300 K were studied by the method of static magnetic susceptibility. An increase in the magnetic moment with increasing temperature up to 1.2 μ_B_ (at 300 K) was found. The magnetic behavior could be explained by an entropy-driven, thermally induced redox isomeric process, but variable temperature spectroscopic studies and DFT calculations did not confirm this assumption, leaving the nature of the magnetic behavior of **2** unresolved. Complex **2** is not immune to the loss of the NO group and easily decomposes into the diamagnetic complex **3** or the paramagnetic complex [Ir(cod)(dpp-bian)](BF_4_)_2_ (**4**). The formation of a rare Ir(II) complex **4** was proven by EPR spectroscopy.

The interesting and unusual findings obtained in this work give us confidence that apparently simple systems in which Ir is coordinated to a redox-active ligand are fraught with surprises. This inspires us to continue research in this area.

## Figures and Tables

**Table 1:** Comparison of selected experimental and calculated geometric parameters (Å) for **1**–**3**.

1		2		3	
Experimental Structure					
Ir—N (dpp-bian)	2.095 (3)	Ir—N (NO)	2.02 (3)	Ir—N (dpp-bian)	2.095 (3)
N—C (dpp-bian)	1.324 (5); 1.448 (5)	Ir—N (dpp-bian)	2.115 (11)	N—C (dpp-bian)	1.298 (5); 1.452 (5)
Ir— Cl	2.4751 (15)	N—C (dpp-bian)	1.296 (16); 1.449 (17);	Ir—C (cod)	2.127 (5)
Ir—C (cod)	2.147 (5); 2.121 (4)	Ir—C (cod)	2.181 (16); 2.241 (16);		
**Calculations for Ground State**					
Ir—N (dpp-bian)	2.103; 2.126	Ir—N (NO)	1.987	Ir—N (dpp-bian)	2.113; 2.109
N—C (dpp-bian)	1.322	Ir—N (dpp-bian)	2.164; 2.131	N—C (dpp-bian)	1.302
Ir— Cl	2.497	N—C (dpp-bian)	1.303; 1.309		
Ir—C (cod)	2.127; 2.143; 2.126; 2.132	Ir—C (cod)	2.330; 2.250; 2.169; 2.221	Ir—C (cod)	2.172; 2.158; 2.176; 2.157

**Table 2:** Fragment contributions to frontier molecular orbitals of [Ir(cod)(dpp-bian)Cl] (**1**), [Ir(cod)(NO)(dpp-bian)]^2+^ (cation of **2**) and [Ir(cod)(dpp-bian)]^+^ (cation of **3**).

[Ir(cod)(dpp-bian)Cl] (1)					
Orbital	E, eV	Ir	dpp-bian	cod	Cl
HOMO-2	−5.460	14.6%	2.9%	3.9%	78.6%
HOMO-1	−5.275	23.0%	15.2%	8.2%	53.6%
HOMO	−4.364	41.7%	34.3%	10.5%	13.5%
LUMO	−3.601	22.7%	64.2%	0.0%	13.1%
LUMO+1	−3.032	0.0%	100.0%	0.0%	0.0%
LUMO+2	−1.700	0.0%	100.0%	0.0%	0.0%
**[Ir(cod)(NO)(dpp-bian)]^2+^ (Cation of 2)**					
Orbital	E, eV	Ir	dpp-bian	cod	NO
HOMO-2	−11.314	1.2%	95.9%	2.9%	0.0%
HOMO-1	−11.201	0.0%	100.0%	0.0%	0.0%
HOMO	−11.157	0.0%	100.0%	0.0%	0.0%
LUMO	−10.001	5.7%	58.7%	4.2%	31.4%
LUMO+1	−9.883	10.5%	9.3%	6.3%	73.9%
LUMO+2	−8.975	24.9%	36.3%	6.0%	32.8%
**[Ir(cod)(dpp-bian)]^+^ (Cation of 3)**					
Orbital	E, eV	Ir	dpp-bian	cod	
HOMO-2	−8.483	28.8%	58.3%	12.9%	
HOMO-1	−8.318	80.8%	14.4%	4.8%	
HOMO	−7.651	86.5%	10.7%	2.8%	
LUMO	−6.804	0.0%	97.6%	2.4%	
LUMO + 1	−5.911	0.0%	99.3%	0.7%	
LUMO + 2	−4.493	5.2%	86.7%	8.1%	

**Table 3:** Calculated diagonal components of g-factor tensor and isotropic g-factor values for [Ir(cod)(dpp-bian)]^2+^ (cation of **4**) compared with experimental values.

	g_xx_	g_yy_	g_zz_	g_iso_
Perturbational SO treatment	2.917	2.757	2.029	2.568
Two-component ZORA SO	2.691	2.598	1.893	2.394
Experiment	2.393	2.393	1.88	2.222

## Data Availability

The crystallographic data have been deposited in the Cambridge Crystallographic Data Centre under the deposition codes CCDC 1030978, 2189440 and 2189441.

## Supplementary Materials

The following supporting information can be downloaded at: https://www.mdpi.com/article/10.3390/ijms241310457/s1. References [38,109] are cited in the supplementary materials.
