# Supplementary material for: Iridium Complexes with BIAN-Type Ligands: Synthesis, Structure and Redox Chemistry
Source: Int J Mol Sci. 2023 Jun 21;24(13):10457. doi: 10.3390/ijms241310457 (PMC10341541; doi:10.3390/ijms241310457)
Supplement: Supplementary file 1 [file ijms-24-10457-s001.zip › ijms-2440232-supplementary.pdf]

Supplementary Materials for

Iridium complexes with BIAN-type ligand: synthesis, structure and redox chemistry

Nikolai F. Romashev [1], Ivan V. Bakaev [1,2], Veronika I. Komlyagina [1,2], Pavel A. Abramov [1,3], Irina V. Mirzaeva [1], Vladimir A. Nadolinny [1], Alexander N. Lavrov [1], Nikolai B. Kompan'kov [1], Artem A. Mikhailov [4], Iakov S. Fomenko [1], Alexander S. Novikov [5,6,*], Maxim N. Sokolov [1] and Artem L. Gushchin [1,*]

[1] Nikolaev Institute of Inorganic Chemistry SB RAS, Novosibirsk 630090, Russia; nikolaj.romashev75@gmail.com (N.F.R.); i.bakaev@g.nsu.ru (I.V.B.); v.komlyagina@g.nsu.ru (V.I.K.); abramov@niic.nsc.ru (P.A.A.); dairdre@gmail.com (I.V.M.); spectr@niic.nsc.ru (V.A.N.); lavrov@niic.nsc.ru (A.N.L.); nmr124@niic.nsc.ru (N.B.K.); fomenko@niic.nsc.ru (I.S.F.); caesar@niic.nsc.ru (M.N.S.)

[2] Department of Natural Sciences, Novosibirsk State University, Novosibirsk 630090, Russia

[3] Research School of Chemistry and Applied Biomedical Sciences, Tomsk Polytechnic University, Tomsk 634034, Russia

[4] Laboratoire de Cristallographie, Résonance Magnétique et Modélisations, Université de Lorraine, CNRS, CRM2, UMR 7036, 54000 Nancy, France; amikhailov@niic.nsc.ru

[5] Institute of Chemistry, Saint Petersburg State University, Saint Petersburg 199034, Russia

[6] Research Institute of Chemistry, Peoples' Friendship University of Russia (RUDN University), Moscow 117198, Russia

* Correspondence: a.s.novikov@spbu.ru (A.S.N.); gushchin@niic.nsc.ru (A.L.G.)

Table of contents

Table S1. Crystal data and structure refinement for **1-3**.

Table S2. Selected geometric parameters (Å) for **1-3**.

Table S3. Comparison of selected calculated bond lengths (Å) and vibrational frequencies (cm^{-1}) for [Ir(cod)(dpp-bian)Cl] (**1**) and [Rh(cod)(dpp-bian)Cl] [1].

Table S4. Comparison of metal and Cl natural electronic configurations and charges on ligands and on metal atoms for [Ir(cod)(dpp-bian)Cl] (**1**) and [Rh(cod)(dpp-bian)Cl] [1].

Table S5. Comparison of calculated characteristic bond lengths (Å) for cation of **2**

Table S6. Comparison of calculated characteristic vibrational frequencies (cm^{-1}) for cation of **2**

Table S7. Topological properties of selected bond critical points for **1**: values of the electron density (rho), Laplacian of electron density (Lap), positively defined kinetic energy density (G), potential energy density (V), total energy density (H), metallicity [2] (M) and elipticity (elip). All values are given in atomic units.

Table S8. Topological properties of selected bond critical points in [Rh(cod)(dpp-bian)Cl] [1]: values of the electron density (rho), Laplacian of electron density (Lap), positively defined kinetic energy density (G), potential energy density (V), total energy density (H), metallicity [2] (M) and elipticity (elip). All values are given in atomic units.

Table S9. Ir natural electron configuration, charge and spin on Ir and ligands for cation of **2**.

Table S10. Topological properties of selected bond critical points for cation of **2**: values of the electron density (rho), Laplacian of electron density (Lap), positively defined kinetic energy density (G), potential energy density (V), total energy density (H), metallicity [2] (M) and elipticity (elip). All values are given in atomic units.

Table S11. Topological properties of selected bond critical points in for cation of **3**: values of the electron density (rho), Laplacian of electron density (Lap), positively defined kinetic energy density (G), potential energy density (V), total energy density (H), metallicity [2] (M) and elipticity (elip). All values are given in atomic units.

Table S12. Topological properties of selected bond critical points for cation of **4**: values of the electron density (rho), Laplacian of electron density (Lap), positively defined kinetic energy density (G), potential energy density (V), total energy density (H), metallicity [2] (M) and elipticity (elip). All values are given in atomic units.

Table S13. Cartesian atomic coordinates for model supramolecular associates used for theoretical analysis of intermolecular π-π interactions in the crystal structures **2** and **3**.

Table S14. Values of the density of all electrons (rho), Laplacian of electron density (Lap) and appropriate λ_2 eigenvalues, energy density (H), potential energy density (V), positively defined

kinetic energy density (G), and electron localization function (ELF) at the bond critical points, corresponding to intermolecular C···C contacts in the crystal structures **2** and **3** (all values are given in atomic units) and their estimated strength (E_{int} = –V/2, in kcal/mol).

Table S15. Parameters of electrochemical experiments performed by cyclic voltammetry at different sweep rates for the first reduction process for complex **1**.

Table S16. Parameters of electrochemical experiments performed by cyclic voltammetry at different sweep rates for the oxidation process for complex **1**.

Table S17. Parameters of electrochemical experiments performed by cyclic voltammetry at different sweep rates for the first reduction process for complex **3**.

Figure S1. UV-vis spectra of **2** in CH_2Cl_2 depending on time. Red line is the UV spectrum of **3** under the same conditions.

Figure S2. ^{1}H NMR spectrum of **1** in $CDCl_3$.

Figure S3. ^{1}H NMR spectrum of **3** in $CDCl_3$.

Figure S4. FT-IR spectrum for **1**.

Figure S5. FT-IR spectrum for **2**.

Figure S6. FT-IR spectrum for **3**.

Figure S7. ORTEP representation (50% probability ellipsoids) of **1**, cation of **2** and cation of **3**.

Figure S8. π-π connected dimers in the crystal packing of **2**.

Figure S9. π-π connected dimers in the crystal packing of **3**.

Figure S10. Crystal packing of **2**.

Figure S11. Crystal packing of **3**.

Figure S12. Contour line diagram of the Laplacian of electron density distribution, bond paths, and selected zero-flux surfaces (left panel), visualization of electron localization function (ELF, center panel) and reduced density gradient (RDG, right panel) analyses for selected intermolecular C···C contacts in the crystal structure **2**. Bond critical points are shown in blue, nuclear critical points – in pale brown, ring critical points – in orange, bond paths are shown as pale brown lines, length units – Å, and the color scale for the ELF and RDG maps is presented in atomic units.

Figure S13. Contour line diagram of the Laplacian of electron density distribution, bond paths, and selected zero-flux surfaces (left panel), visualization of electron localization function (ELF, center panel) and reduced density gradient (RDG, right panel) analyses for selected intermolecular C···C contacts in the crystal structure **3**. Bond critical points are shown in blue,

nuclear critical points – in pale brown, ring critical points – in orange, bond paths are shown as pale brown lines, length units – Å, and the color scale for the ELF and RDG maps is presented in atomic units.

Figure S14. CV of **3** in CH_2Cl_2 in the -1.5–1.8 V region at potential scan rate of 100 mV/s (blue spectrum – reduction part, red spectrum – oxidation part).

Figure S15. CVs of **1** in CH_2Cl_2 (oxidation process), recorded at various potential scan rates (50-200 mV/s).

Figure S16. Dependence of the cathodic current on the square root of the sweep rate for the oxidation process for **1**.

Figure S17. Dependence of the anodic current on the square root of the sweep rate for the oxidation process for **1**.

Figure S18. CVs of **1** in CH_2Cl_2 (first reduction process), recorded at various potential scan rates (50-200 mV/s).

Figure S19. Dependence of the cathodic current on the square root of the sweep rate for the first reduction process for **1**.

Figure S20. Dependence of the anodic current on the square root of the sweep rate for the first reduction process for **1**.

Figure S21. CVs of **3** in CH_2Cl_2 (first reduction process), recorded at various potential scan rates (50-200 mV/s).

Figure S22. Dependence of the cathodic current on the square root of the sweep rate for the first reduction process for **3**.

Figure S23. Dependence of the anodic current on the square root of the sweep rate for the first reduction process for **3**.

Figure S24. Dependence of the FT-IR spectrum of **2** on temperature.

Figure S25. Dependence of the diffuse reflectance spectrum of **2** on temperature.

Figure S26. Electronic energy levels for $[Ir(cod)(dpp\text{-}bian)]^{2+}$ (cation of **4**) and its fragments.

Table S1. Crystal data and structure refinement for **1-3**.

	**(1)**	**(2)**	**(3)**
Chemical formula	$C_{44}H_{52}ClIrN_2$	$C_{44.40}H_{52.80}BCl_{0.80}F_4IrN_3O$	$C_{44}H_{52}BF_4IrN_2$
M_r	836.52	951.86	887.88
Crystal system, space group	Orthorhombic, *Pnma*	Monoclinic, $P2_1/n$	Tetragonal, $P4_2/mbc$
Temperature (K)	150	86	150
a, *b*, *c* (Å)	18.5911 (7), 19.4115 (8), 10.2299 (5)	11.2382 (18), 22.668 (3), 20.330 (3)	20.1791 (4), 20.1791 (4), 19.1826 (5)
α, β, γ (°)	90, 90, 90	90, 97.427 (5), 90	90, 90, 90
V (Å^3)	3691.8 (3)	5135.7 (14)	7811.1 (4)
Z	4	4	8
Radiation type	Cu *K*a	Mo *K*a	Mo *K*a
μ (mm^{-1})	7.91	2.69	3.47
Crystal size (mm)	0.06 × 0.04 × 0.02	0.12 × 0.12 × 0.07	0.18 × 0.14 × 0.11
Diffractometer	Bruker Apex Duo	Bruker D8 Venture diffractometer	Bruker D8 Venture diffractometer
Absorption correction	Multi-scan *SADABS* (Bruker-AXS, 2004)	Multi-scan *SADABS* 2016/2: Krause, L., Herbst-Irmer, R., Sheldrick G.M. & Stalke D., J. Appl. Cryst. 48 (2015) 3-10	Multi-scan *SADABS* 2016/2: Krause, L., Herbst-Irmer, R., Sheldrick G.M. & Stalke D., J. Appl. Cryst. 48 (2015) 3-10
T_{min}, T_{max}	0.640, 0.753	0.602, 0.745	0.600, 0.746
No. of measured, independent and observed [$I > 2\sigma(I)$] reflections	15939, 3454, 2936	32478, 7275, 4452	36785, 5035, 3453
R_{int}	0.039	0.164	0.084
θ_{max} (°)	69.9	23.3	28.7
$(\sin \theta/\lambda)_{max}$ (Å^{-1})	0.609	0.556	0.676
Range of *h*, *k*, *l*	$-20 \le h \le 21$, $-23 \le k \le 17$, $-12 \le h \le 11$	$-12 \le h \le 12$, $-25 \le k \le 25$, $-22 \le l \le 21$	$-24 \le h \le 25$, $-25 \le k \le 24$, $-22 \le l \le 25$
$R[F^2 > 2\sigma(F^2)]$, $wR(F^2)$, S	0.032, 0.075, 1.04	0.071, 0.239, 0.79	0.041, 0.106, 1.01
No. of reflections, parameters, restraints	3454, 227, 12	7275, 492, 12	5035, 244, 18
Weighting scheme	$w = 1/[\sigma^2(F_o^2) + (0.031P)^2 + 7.9633P]$ where $P = (F_o^2 + 2F_c^2)/3$	$w = 1/[\sigma^2(F_o^2) + (0.2P)^2]$ where $P = (F_o^2 + 2F_c^2)/3$	$w = 1/[\sigma^2(F_o^2) + (0.0552P)^2]$ where $P = (F_o^2 + 2F_c^2)/3$
$\Delta\rho_{max}$, $\Delta\rho_{min}$ (e Å^{-3})	1.33, -1.04	1.43, -1.49	1.32, -1.27

Computer programs: *APEX2* (Bruker-AXS, 2004), *APEX3* (Bruker-AXS, 2016), *SAINT* (Bruker-AXS, 2004), *SAINT* (Bruker-AXS, 2016), *SHELXS2014* (Sheldrick, 2014), *SHELXT* 2014/5 (Sheldrick, 2014), *SHELXL2014* (Sheldrick, 2014), *SHELXL2017*/1 (Sheldrick, 2017), ShelXle (Hübschle, 2011), CIFTAB-2014 (Sheldrick, 2014).

Table S2. Selected geometric parameters (Å) for **1-3**.

**(1)**		**(2)**		**(3)**	
Ir1—N1^{i}	2.095 (3)	N1B—Ir1	2.02 (3)	N1—Ir1	2.095 (3)
Ir1—N1	2.095 (3)	N1A—Ir1	1.91 (5)	C5—N1	1.298 (5)
N1—C01	1.324 (5)	N2—Ir1	2.115 (11)	C12—N1	1.452 (5)
N1—C11	1.448 (5)	N3—Ir1	2.127 (10)	C2—Ir1	2.127 (5)
Ir1—Cl1	2.4751 (15)	C1—N3	1.296 (16)	C3—Ir1	2.127 (5)
Ir1—C112^{i}	2.147 (5)	C8—N2	1.302 (17)		
Ir1—C112	2.147 (5)	C21—N2	1.449 (17)		
Ir1—C113^{i}	2.121 (4)	C32—N3	1.472 (17)		
Ir1—C113	2.121 (4)	C37—Ir1	2.181 (16)		
		C40—Ir1	2.241 (16)		
		C43—Ir1	2.235 (16)		
		C44—Ir1	2.184 (15)		

Symmetry code(s): (i) x, $-y+3/2$, z.

Table S3. Comparison of selected calculated bond lengths (Å) and vibrational frequencies (cm^{-1}) for [Ir(cod)(dpp-bian)Cl] (**1**) and [Rh(cod)(dpp-bian)Cl] [1].

	Ir/Rh-Cl	Ir/Rh-N	C=C (dpp-bian)	C-N	ν(C=C) (dpp-bian)	ν_{asym}(C-N)	ν_{sym}(C-N)
[(dpp-bian) IrCl(cod)] ground	2.497	2.103; 2.126	1.442	1.322	1312	1545	1524
[(dpp-bian) IrCl(cod)] excited	2.608	2.107	1.435	1.336	1313	1468	1556
[(dpp-bian) RhCl(cod)]	2.574	2.131; 2.144	1.455	1.317; 1.318	1295	1549	1524

Table S4. Comparison of metal and Cl natural electronic configurations and charges on ligands and on metal atoms for [Ir(cod)(dpp-bian)Cl] (**1**) and [Rh(cod)(dpp-bian)Cl] [1].

	[Ir(cod)(dpp-bian)Cl]		[Rh(cod)(dpp-bian)Cl]	
Ir/Rh Natural configuration	[core]6s(0.35)5d(7.76)6p(0.01)6d(0.02)		[core]5s(0.22)4d(8.03)5p(0.01)5d(0.02)6p(0.01)	
Cl Natural configuration	[core]3s(1.96)3p(5.69)		[core]3s(1.96)3p(5.65)	
	NPA	AIM	NPA	AIM
Ir/Rh	0.8503	0.8893	0.7141	0.7734
Cl	-0.6574	-0.5467	-0.6163	-0.6012
dpp-bian	-0.2473	-0.1866	-0.2911	-0.1324
cod	0.0544	-0.1560	0.19329	-0.0398

Table S5. Comparison of calculated characteristic bond lengths (Å) for cation of **2**

	Ir-N (NO)	<Ir-N-O	N=O	Ir-N (dpp-bian)	C=C (dpp-bian)	C-N
[(dpp-bian) $IrNO(cod)]^{2+}$ **exp**.	1.914 (v1)/ 2.019 (v2)	127.0 (v1)/ 123.8 (v2)	1.247 (v1)/ 1.096 (v2)	2.127; 2.116	1.503	1.295; 1.301
[(dpp-bian) $IrNO(cod)]^{2+}$ ground -v1	1.987	127.3	1.167	2.164/ 2.131	1.508	1.303; 1.309
[(dpp-bian) $IrNO(cod)]^{2+}$ ground-v2	1.968	129.9	1.166	2.122; 2.210	1.509	1.302; 1.312
[(dpp-bian) $IrNO(cod)]^{2+}$ excited-v1	2.018	129.0	1.172	2.147; 2.086	1.437	1.343; 1.340
[(dpp-bian) $IrNO(cod)]^{2+}$ excited-v2	2.002	131.5	1.172	2.077; 2.177	1.438	1.345; 1.337

Table S6. Comparison of calculated characteristic vibrational frequencies (cm^{-1}) for cation of **2**

	ν(C=C) (dpp-bian)	ν_{asym}(C-N)	ν_{sym}(C-N)	ν(N=O)	ν(<Ir-N-O)
[(dpp-bian) $IrNO(cod)]^{2+}$ ground -v1	1289	1580	1557	1743	521
[(dpp-bian) $IrNO(cod)]^{2+}$ ground-v2	1288	1580	1545	1755	525
[(dpp-bian) $IrNO(cod)]^{2+}$ excited-v1	1423	1438	1474	1718	478
[(dpp-bian) $IrNO(cod)]^{2+}$ excited-v2	1424	1440	1474	1719	480

Table S7. Topological properties of selected bond critical points for **1**: values of the electron density (rho), Laplacian of electron density (Lap), positively defined kinetic energy density (G), potential energy density (V), total energy density (H), metallicity [2] (M) and elipticity (elip). All values are given in atomic units.

BOND	rho	Lap	V	G	H	M	elip
Ir-N	0.1037	0.3726	-0.1624	0.1278	-0.0346	4.2310	0.0905
Ir-N	0.0989	0.3438	-0.1501	0.1180	-0.0321	4.2376	0.0538
Ir-C	0.1085	0.1455	-0.1539	0.0951	-0.0588	11.6918	0.3038
Ir-C	0.1116	0.1438	-0.1606	0.0983	-0.0623	12.4035	0.1462
Ir-C	0.1124	0.1384	-0.1618	0.0982	-0.0636	13.0287	0.2624
Ir-C	0.1107	0.1543	-0.1593	0.0989	-0.0604	11.3924	0.1758
C=C (cod)	0.2837	-0.6646	-0.6479	0.2409	-0.4071	-12.6983	0.2023
C=C (cod)	0.2808	-0.6494	-0.6373	0.2375	-0.3998	-12.7765	0.2038
C=C (dpp-bian)	0.2868	-0.7173	-0.6566	0.2387	-0.4180	-11.9849	0.1805
C-N	0.3428	-0.9589	-0.8843	0.3223	-0.5620	-12.0672	0.2301
C-N	0.3423	-0.9543	-0.8823	0.3219	-0.5604	-12.0946	0.2321
Ir-Cl	0.0689	0.1559	-0.0795	0.0592	-0.0203	5.1212	0.0423

Table S8. Topological properties of selected bond critical points in [Rh(cod)(dpp-bian)Cl] [1]: values of the electron density (rho), Laplacian of electron density (Lap), positively defined kinetic energy density (G), potential energy density (V), total energy density (H), metallicity [2] (M) and elipticity (elip). All values are given in atomic units.

BOND	rho	Lap	V	G	H	M	elip
Rh-N	0.0850	0.3305	-0.1219	0.1023	-0.0196	3.4253	0.0601
Rh-N	0.0874	0.3498	-0.1280	0.1077	-0.0203	3.3906	0.0759
Rh-C	0.0987	0.1707	-0.1353	0.0890	-0.0463	8.5164	0.4625
Rh-C	0.0976	0.1789	-0.1337	0.0892	-0.0445	7.9672	0.3911
Rh-C	0.0923	0.1775	-0.1230	0.0837	-0.0393	7.3159	0.6877
Rh-C	0.0967	0.1675	-0.1309	0.0864	-0.0445	8.3806	0.2943
C=C (cod)	0.2916	-0.7036	-0.6777	0.2509	-0.4268	-12.5573	0.2127
C=C (cod)	0.2874	-0.6811	-0.6618	0.2458	-0.4160	-12.6592	0.2121
C=C (dpp-bian)	0.2802	-0.6883	-0.6316	0.2298	-0.4019	-12.0113	0.1622
C-N	0.3462	-0.9698	-0.8994	0.3285	-0.5709	-12.1283	0.2349
C-N	0.3470	-0.9747	-0.9028	0.3295	-0.5732	-12.1138	0.2328
Rh-Cl	0.0525	0.1399	-0.0539	0.0444	-0.0095	3.6259	0.0391

Table S9. Ir natural electron configuration, charges and spins on Ir and ligands for cation of **2**.

	v1 ground		v2 ground	
Ir Natural configuration	[core]6s(0.36)5d(7.72)6p(0.01)7s(0.01)6d(0.02)		[core]6s(0.36)5d(7.71)6p(0.01)7s(0.01)6d(0.02)7p(0.01)	
	NPA	AIM	NPA	AIM
Ir	0.8851	0.9069	0.89423	0.9294
NO	0.0608	-0.0578	0.07169	-0.0438
dpp-bian	0.70443	0.7355	0.68605	0.7057
cod	0.3497	0.4154	0.34803	0.4087

	v1 excited				v2 excited			
Ir Natural configuration	α — [core]6s(0.17)5d(3.86)6d(0.01)<br>β — [core]6s(0.18)5d(3.86)6d(0.01)				α — [core]6s(0.17)5d(3.85)6d(0.01)<br>β — [core]6s(0.18)5d(3.86)6d(0.01)			
	NPA	NPA Spin	AIM	AIM Spin	NPA	NPA Spin	AIM	AIM Spin
Ir	0.9020	0.0017	0.9284	0.0368	0.9043	-0.0037	0.9164	0.032
NO	0.0131	0.2940	-0.0894	0.2809	0.0122	0.3354	-0.0856	0.3199
dpp-bian	0.8116	1.6007	0.8281	1.5850	0.8012	1.5675	0.8182	1.5532
cod	0.2733	0.1036	0.3329	0.0973	0.2824	0.1009	0.351	0.0949

Table S10. Topological properties of selected bond critical points for cation of **2**: values of the electron density (rho), Laplacian of electron density (Lap), positively defined kinetic energy density (G), potential energy density (V), total energy density (H), metallicity [2] (M) and elipticity (elip). All values are given in atomic units.

BOND	rho	Lap	V	G	H	M	elip
Ir-N(=O)	0.1927	0.4768	-0.4090	0.2641	-0.1449	9.2944	0.1213
Ir-N	0.0927	0.2753	-0.1320	0.1004	-0.0316	4.7545	0.1478
Ir-N	0.1055	0.3189	-0.1618	0.1208	-0.0410	5.0879	0.0710
Ir-C	0.0883	0.1493	-0.1129	0.0751	-0.0378	8.0782	0.9725
Ir-C	0.0962	0.1527	-0.1287	0.0834	-0.0452	9.1101	0.1941
Ir-C	0.1127	0.0760	-0.1574	0.0882	-0.0692	23.8694	0.1032
N=O	0.4003	-0.5654	-1.2015	0.5300	-0.6714	-26.4981	0.0468
C=C (cod)	0.2933	-0.7126	-0.6840	0.2529	-0.4311	-12.5192	0.2060
C=C (cod)	0.2614	-0.5982	-0.5639	0.2072	-0.3567	-12.3110	0.0634
C=C (dpp-bian)	0.2552	-0.5779	-0.5415	0.1985	-0.3430	-12.2437	0.0945
C-N	0.3536	-1.0114	-0.9311	0.3392	-0.5920	-12.0482	0.2155
C-N	0.3530	-1.0041	-0.9290	0.3390	-0.5900	-12.1028	0.2124

Table S11. Topological properties of selected bond critical points for cation of **3**: values of the electron density (rho), Laplacian of electron density (Lap), positively defined kinetic energy density (G), potential energy density (V), total energy density (H), metallicity [2] (M) and elipticity (elip). All values are given in atomic units.

BOND	rho	Lap	V	G	H	M	elip
Ir-N	0.1036	0.3681	-0.1620	0.1270	-0.0350	4.2805	0.1817
Ir-N	0.1017	0.3545	-0.1568	0.1227	-0.0341	4.3079	0.1804
Ir-C	0.1103	0.1545	-0.1585	0.0985	-0.0599	11.3046	0.2190
Ir-C	0.1042	0.1672	-0.1464	0.0941	-0.0523	9.5030	0.5049
Ir-C	0.1053	0.1700	-0.1489	0.0957	-0.0532	9.5088	0.3660
Ir-C	0.1084	0.1516	-0.1543	0.0961	-0.0582	11.2067	0.3361
C=C (cod)	0.2890	-0.6882	-0.6681	0.2480	-0.4201	-12.6489	0.2173
C=C (cod)	0.2894	-0.6905	-0.6696	0.2485	-0.4211	-12.6362	0.2173
C=C (dpp-bian)	0.2680	-0.6389	-0.5864	0.2134	-0.3731	-12.0148	0.1137
C-N	0.3580	-1.0148	-0.9517	0.3490	-0.6027	-12.2536	0.2255
C-N	0.3570	-1.0090	-0.9475	0.3476	-0.5999	-12.2692	0.2293

Table S12. Topological properties of selected bond critical points for cation of **4**: values of the electron density (rho), Laplacian of electron density (Lap), positively defined kinetic energy density (G), potential energy density (V), total energy density (H), metallicity [2] (M) and elipticity (elip). All values are given in atomic units.

BOND	rho	Lap	V	G	H	M	elip
Ir-N	0.1045	0.3369	-0.1611	0.1227	-0.0384	4.7384	0.1050
Ir-N	0.1055	0.3390	-0.1636	0.1242	-0.0394	4.7897	0.1104
Ir-C	0.1036	0.1433	-0.1433	0.0895	-0.0537	10.9995	0.3273
Ir-C	0.1016	0.1503	-0.1397	0.0886	-0.0510	10.1503	0.3790
Ir-C	0.1008	0.1522	-0.1381	0.0881	-0.0500	9.8871	0.4071
Ir-C	0.1039	0.1421	-0.1438	0.0897	-0.0541	11.1411	0.3092
C=C (cod)	0.2895	-0.6943	-0.6697	0.2481	-0.4216	-12.5746	0.2004
C=C (cod)	0.2894	-0.6938	-0.6693	0.2479	-0.4214	-12.5764	0.2002
C=C (dpp-bian)	0.2617	-0.6122	-0.5638	0.2054	-0.3584	-12.0514	0.0936
C-N	0.3582	-1.0137	-0.9531	0.3498	-0.6033	-12.2824	0.2160
C-N	0.3582	-1.0119	-0.9533	0.3501	-0.6031	-12.3049	0.2173

Table S13. Cartesian atomic coordinates for model supramolecular associates used for theoretical analysis of intermolecular π-π interactions in the crystal structures **2** and **3**.

Atom	X	Y	Z
		**2**	
C	1.518120	8.314622	9.898284
C	1.964771	9.404953	10.670391
C	2.155167	9.611232	12.063408
H	1.996447	8.916412	12.692342
C	2.606633	10.946377	12.496836
H	2.731569	11.118019	13.422053
C	2.848582	11.932435	11.597725
H	3.149336	12.773146	11.920175
C	2.672651	11.762425	10.188580
C	2.223605	10.454482	9.805551
C	1.487542	8.779316	8.468980
C	1.925016	10.157531	8.440757
C	2.093643	11.139055	7.448913
H	1.920129	10.956260	6.532283
C	2.536381	12.415264	7.880325
H	2.653925	13.097774	7.231130
C	2.808307	12.712214	9.216895
H	3.091708	13.587834	9.453406
C	-1.669573	10.368343	7.686794
H	-0.795604	10.523234	8.100808
H	-1.873162	11.099024	7.067173
H	-2.358767	10.328470	8.383040
C	-1.643096	9.033198	6.914687
H	-1.457341	8.301928	7.571542

C	-2.980255	8.738514	6.249426
H	-3.689155	8.733890	6.926400
H	-3.172186	9.429163	5.580899
H	-2.941491	7.862486	5.811623
C	-0.506289	9.051332	5.892604
C	-0.663167	9.574963	4.664894
H	-1.512642	9.929219	4.429472
C	0.346061	9.622566	3.733528
H	0.198386	10.029298	2.888263
C	1.589782	9.067200	4.037936
H	2.278022	9.088417	3.384790
C	1.851129	8.473298	5.291853
C	0.772823	8.482366	6.227251
C	3.523952	6.648524	4.701181
H	4.415260	6.309207	4.926402
H	2.856605	5.950531	4.871448
H	3.499155	6.899572	3.754534
C	3.201278	7.911132	5.590212
H	3.187995	7.605545	6.541718
C	4.316404	8.940259	5.467240
H	5.175826	8.517750	5.673430
H	4.336703	9.292905	4.552687
H	4.154101	9.674680	6.096073
C	-1.620654	9.085334	11.432418
H	-0.742290	9.460558	11.213204
H	-2.297683	9.470872	10.836747
H	-1.844674	9.297802	12.361848

C	-1.586888	7.575646	11.250983
H	-1.407917	7.392012	10.283330
C	-2.936127	6.977210	11.583613
H	-2.902073	6.005592	11.462637
H	-3.163917	7.183557	12.513770
H	-3.616926	7.356650	10.988668
C	-0.474863	6.974944	12.031153
C	-0.665162	6.619056	13.361676
H	-1.502328	6.805682	13.771255
C	0.318720	6.004753	14.107575
H	0.154447	5.748310	15.007049
C	1.576780	5.764472	13.508840
H	2.251932	5.330879	14.017523
C	0.809727	6.736930	11.539263
C	1.853787	6.136228	12.228715
C	3.238178	5.914081	11.712634
H	3.261822	6.256776	10.774837
C	4.303529	6.657592	12.488772
H	5.183361	6.469017	12.100784
H	4.127416	7.621480	12.444018
H	4.290525	6.367668	13.425037
C	3.588564	4.359056	11.654171
H	4.499659	4.243586	11.313013
H	3.524613	3.976103	12.553484
H	2.956269	3.905130	11.059145
C	-0.878322	5.687401	6.729221
H	-0.968586	6.430368	6.064786

C	-2.189727	5.204573	7.331988
H	-2.784801	5.989906	7.429580
H	-2.615981	4.594328	6.680495
C	-2.142901	4.497331	8.662511
H	-2.255176	3.524783	8.513412
H	-2.900168	4.807361	9.219032
C	0.181274	4.862286	6.523594
H	0.761322	5.133055	5.754592
C	0.223368	3.368465	6.830018
H	-0.648818	2.976331	6.575182
H	0.913573	2.954298	6.254385
C	0.516803	2.980842	8.277465
H	-0.061308	2.213916	8.519338
H	1.455719	2.679766	8.338428
C	0.305778	4.037171	9.241087
H	0.815838	3.910570	10.091452
C	-0.871131	4.730812	9.398330
H	-1.028473	4.998883	10.349473
N	2.385482	5.537792	7.908548
N	0.984398	7.983670	7.569869
N	1.040166	7.135886	10.142214
Ir	0.498581	6.087265	8.372416
O	2.910833	4.857752	7.499311
C	7.092165	14.353378	10.261154
C	6.645514	13.263047	9.489048
C	6.455119	13.056768	8.096031
H	6.613839	13.751588	7.467096

C	6.003652	11.721623	7.662603
H	5.878717	11.549981	6.737385
C	5.761703	10.735565	8.561714
H	5.460949	9.894854	8.239263
C	5.937634	10.905575	9.970858
C	6.386680	12.213518	10.353888
C	7.122743	13.888684	11.690458
C	6.685270	12.510469	11.718682
C	6.516643	11.528945	12.710526
H	6.690156	11.711740	13.627156
C	6.073904	10.252736	12.279114
H	5.956361	9.570226	12.928308
C	5.801978	9.955786	10.942543
H	5.518578	9.080166	10.706033
C	10.279858	12.299657	12.472645
H	9.405890	12.144766	12.058630
H	10.483447	11.568976	13.092265
H	10.969053	12.339530	11.776398
C	10.253381	13.634802	13.244751
H	10.067627	14.366072	12.587896
C	11.590540	13.929486	13.910013
H	12.299440	13.934110	13.233039
H	11.782471	13.238837	14.578540
H	11.551777	14.805514	14.347815
C	9.116575	13.616668	14.266835
C	9.273452	13.093037	15.494545
H	10.122928	12.738781	15.729966

C	8.264224	13.045434	16.425911
H	8.411899	12.638702	17.271176
C	7.020504	13.600800	16.121503
H	6.332264	13.579583	16.774649
C	6.759156	14.194702	14.867586
C	7.837462	14.185634	13.932188
C	5.086334	16.019476	15.458258
H	4.195026	16.358793	15.233036
H	5.753680	16.717469	15.287991
H	5.111130	15.768428	16.404904
C	5.409008	14.756868	14.569226
H	5.422290	15.062455	13.617721
C	4.293881	13.727741	14.692199
H	3.434459	14.150250	14.486008
H	4.273582	13.375095	15.606752
H	4.456184	12.993320	14.063365
C	10.230939	13.582666	8.727021
H	9.352575	13.207442	8.946235
H	10.907968	13.197128	9.322692
H	10.454959	13.370198	7.797590
C	10.197173	15.092354	8.908456
H	10.018203	15.275988	9.876109
C	11.546413	15.690790	8.575825
H	11.512358	16.662408	8.696802
H	11.774203	15.484443	7.645669
H	12.227211	15.311350	9.170771
C	9.085148	15.693056	8.128286

C	9.275448	16.048944	6.797763
H	10.112613	15.862318	6.388183
C	8.291566	16.663247	6.051863
H	8.455839	16.919690	5.152390
C	7.033506	16.903528	6.650599
H	6.358353	17.337121	6.141916
C	7.800558	15.931070	8.620176
C	6.756499	16.531772	7.930723
C	5.372107	16.753919	8.446805
H	5.348463	16.411224	9.384602
C	4.306757	16.010408	7.670666
H	3.426924	16.198983	8.058655
H	4.482869	15.046520	7.715420
H	4.319760	16.300332	6.734402
C	5.021721	18.308944	8.505267
H	4.110626	18.424414	8.846425
H	5.085672	18.691897	7.605955
H	5.654016	18.762870	9.100293
C	9.488607	16.980599	13.430218
H	9.578872	16.237632	14.094653
C	10.800012	17.463427	12.827451
H	11.395086	16.678094	12.729859
H	11.226267	18.073672	13.478943
C	10.753186	18.170669	11.496928
H	10.865462	19.143217	11.646027
H	11.510453	17.860639	10.940406
C	8.429012	17.805714	13.635844

H	7.848964	17.534945	14.404846
C	8.386917	19.299535	13.329421
H	9.259103	19.691669	13.584256
H	7.696712	19.713702	13.905053
C	8.093483	19.687158	11.881973
H	8.671594	20.454084	11.640100
H	7.154566	19.988234	11.821011
C	8.304507	18.630829	10.918352
H	7.794447	18.757430	10.067987
C	9.481416	17.937188	10.761108
H	9.638758	17.669117	9.809966
N	6.224803	17.130208	12.250891
N	7.625887	14.684330	12.589569
N	7.570120	15.532114	10.017225
Ir	8.111704	16.580735	11.787022
O	5.699452	17.810248	12.660127

3

C	19.117679	12.712833	10.287628
H	19.658116	13.482585	10.593917
H	19.576774	11.890333	10.593917
C	17.771733	12.781442	10.976284
H	17.709965	12.221976	11.802900
C	16.922193	13.885239	10.976284
H	16.366864	13.960446	11.804895
C	17.184522	15.192844	10.320239
H	16.501439	15.837466	10.629539
H	18.065541	15.523176	10.629462

C	13.931045	10.860392	8.844905
C	12.819782	10.029013	8.398142
C	12.365752	9.577001	7.187720
H	12.769395	9.871374	6.379864
C	11.288189	8.670959	7.160865
H	10.961388	8.367486	6.322220
C	10.690887	8.206840	8.317575
H	9.971159	7.591337	8.261505
C	11.144917	8.644726	9.591300
C	12.208355	9.538661	9.591300
C	15.087913	11.310386	6.817496
C	14.409895	12.180105	5.956197
C	14.637919	12.002529	4.590396
H	14.209174	12.582476	3.971949
C	15.455173	11.023842	4.120422
H	15.572111	10.917700	3.183909
C	16.113011	10.188428	4.989394
H	16.690073	9.518744	4.641882
C	15.955614	10.303448	6.374378
C	18.076438	9.034183	6.899981
H	18.055431	8.389985	6.162353
H	18.560272	8.646381	7.659171
H	18.531194	9.851840	6.606986
C	16.653811	9.369156	7.321998
H	16.704380	9.824538	8.211457
C	15.854719	8.069622	7.513824
H	14.956184	8.285236	7.842250

H	16.311392	7.497121	8.163673
H	15.784193	7.600559	6.656228
C	12.036833	12.979197	5.927423
H	12.032737	13.060801	4.951662
H	11.421875	13.637419	6.313799
H	11.752389	12.077111	6.182456
C	13.443316	13.233454	6.451108
H	13.418859	13.190694	7.450215
C	13.824701	14.652045	6.042519
H	14.697710	14.879624	6.424176
H	13.149146	15.280623	6.377006
H	13.868490	14.710947	5.065875
N	14.923655	11.452245	8.252930
Ir	16.191508	12.447074	9.591300
C	19.117679	12.712833	8.894972
H	19.658116	13.482585	8.588683
H	19.576774	11.890333	8.588683
C	17.771733	12.781442	8.206316
H	17.709965	12.221976	7.379700
C	16.922193	13.885239	8.206316
H	16.366864	13.960446	7.377705
C	17.184522	15.192844	8.862361
H	16.501439	15.837466	8.553061
H	18.065541	15.523176	8.553138
C	13.931045	10.860392	10.337695
C	12.819782	10.029013	10.784458
C	12.365752	9.577001	11.994880

H	12.769395	9.871374	12.802736
C	11.288189	8.670959	12.021735
H	10.961388	8.367486	12.860380
C	10.690887	8.206840	10.865025
H	9.971159	7.591337	10.921095
C	15.087913	11.310386	12.365104
C	14.409895	12.180105	13.226403
C	14.637919	12.002529	14.592204
H	14.209174	12.582476	15.210651
C	15.455173	11.023842	15.062178
H	15.572111	10.917700	15.998691
C	16.113011	10.188428	14.193206
H	16.690073	9.518744	14.540718
C	15.955614	10.303448	12.808222
C	18.076438	9.034183	12.282619
H	18.055431	8.389985	13.020247
H	18.560272	8.646381	11.523429
H	18.531194	9.851840	12.575614
C	16.653811	9.369156	11.860602
H	16.704380	9.824538	10.971143
C	15.854719	8.069622	11.668776
H	14.956184	8.285236	11.340350
H	16.311392	7.497121	11.018927
H	15.784193	7.600559	12.526372
C	12.036833	12.979197	13.255177
H	12.032737	13.060801	14.230938
H	11.421875	13.637419	12.868801

H	11.752389	12.077111	13.000144
C	13.443316	13.233454	12.731492
H	13.418859	13.190694	11.732385
C	13.824701	14.652045	13.140081
H	14.697710	14.879624	12.758424
H	13.149146	15.280623	12.805594
H	13.868490	14.710947	14.116725
N	14.923655	11.452245	10.929670
C	1.061421	7.466267	10.287628
H	0.520984	6.696515	10.593917
H	0.602326	8.288767	10.593917
C	2.407367	7.397658	10.976284
H	2.469135	7.957124	11.802900
C	3.256907	6.293861	10.976284
H	3.812236	6.218654	11.804895
C	2.994578	4.986256	10.320239
H	3.677661	4.341634	10.629539
H	2.113559	4.655924	10.629462
C	6.248055	9.318708	8.844905
C	7.359318	10.150087	8.398142
C	7.813348	10.602099	7.187720
H	7.409705	10.307726	6.379864
C	8.890911	11.508141	7.160865
H	9.217712	11.811614	6.322220
C	9.488213	11.972260	8.317575
H	10.207941	12.587763	8.261505
C	9.034183	11.534374	9.591300

C	7.970745	10.640439	9.591300
C	5.091187	8.868714	6.817496
C	5.769205	7.998995	5.956197
C	5.541181	8.176571	4.590396
H	5.969926	7.596624	3.971949
C	4.723927	9.155258	4.120422
H	4.606989	9.261400	3.183909
C	4.066089	9.990672	4.989394
H	3.489027	10.660356	4.641882
C	4.223486	9.875652	6.374378
C	2.102662	11.144917	6.899981
H	2.123669	11.789115	6.162353
H	1.618828	11.532719	7.659171
H	1.647906	10.327260	6.606986
C	3.525289	10.809944	7.321998
H	3.474720	10.354562	8.211457
C	4.324381	12.109478	7.513824
H	5.222916	11.893864	7.842250
H	3.867708	12.681979	8.163673
H	4.394907	12.578541	6.656228
C	8.142267	7.199903	5.927423
H	8.146363	7.118299	4.951662
H	8.757225	6.541681	6.313799
H	8.426711	8.101989	6.182456
C	6.735784	6.945646	6.451108
H	6.760241	6.988406	7.450215
C	6.354399	5.527055	6.042519

H	5.481390	5.299476	6.424176
H	7.029954	4.898477	6.377006
H	6.310610	5.468153	5.065875
N	5.255445	8.726855	8.252930
Ir	3.987592	7.732026	9.591300
C	1.061421	7.466267	8.894972
H	0.520984	6.696515	8.588683
H	0.602326	8.288767	8.588683
C	2.407367	7.397658	8.206316
H	2.469135	7.957124	7.379700
C	3.256907	6.293861	8.206316
H	3.812236	6.218654	7.377705
C	2.994578	4.986256	8.862361
H	3.677661	4.341634	8.553061
H	2.113559	4.655924	8.553138
C	6.248055	9.318708	10.337695
C	7.359318	10.150087	10.784458
C	7.813348	10.602099	11.994880
H	7.409705	10.307726	12.802736
C	8.890911	11.508141	12.021735
H	9.217712	11.811614	12.860380
C	9.488213	11.972260	10.865025
H	10.207941	12.587763	10.921095
C	5.091187	8.868714	12.365104
C	5.769205	7.998995	13.226403
C	5.541181	8.176571	14.592204
H	5.969926	7.596624	15.210651

C	4.723927	9.155258	15.062178
H	4.606989	9.261400	15.998691
C	4.066089	9.990672	14.193206
H	3.489027	10.660356	14.540718
C	4.223486	9.875652	12.808222
C	2.102662	11.144917	12.282619
H	2.123669	11.789115	13.020247
H	1.618828	11.532719	11.523429
H	1.647906	10.327260	12.575614
C	3.525289	10.809944	11.860602
H	3.474720	10.354562	10.971143
C	4.324381	12.109478	11.668776
H	5.222916	11.893864	11.340350
H	3.867708	12.681979	11.018927
H	4.394907	12.578541	12.526372
C	8.142267	7.199903	13.255177
H	8.146363	7.118299	14.230938
H	8.757225	6.541681	12.868801
H	8.426711	8.101989	13.000144
C	6.735784	6.945646	12.731492
H	6.760241	6.988406	11.732385
C	6.354399	5.527055	13.140081
H	5.481390	5.299476	12.758424
H	7.029954	4.898477	12.805594
H	6.310610	5.468153	14.116725
N	5.255445	8.726855	10.929670

Table S14. Values of the density of all electrons (rho), Laplacian of electron density (Lap) and appropriate λ_2 eigenvalues, energy density (H), potential energy density (V), positively defined kinetic energy density (G), and electron localization function (ELF) at the bond critical points, corresponding to intermolecular C···C contacts in the crystal structures **2** and **3** (all values are given in atomic units) and their estimated strength (E_{int} = −V/2, in kcal/mol).

Contact	rho	Lap	λ_2	H	V	G	ELF	E_{int}
**2**								
3.543 Å	0.005	0.014	-0.005	0.001	-0.002	0.003	0.020	0.6
3.691 Å	0.006	0.018	-0.006	0.001	-0.002	0.003	0.021	0.6
3.700 Å	0.005	0.014	-0.005	0.001	-0.002	0.003	0.020	0.6
**3**								
3.578 Å	0.004	0.012	-0.004	0.000	-0.002	0.002	0.016	0.6
3.714 Å	0.003	0.009	-0.003	0.001	-0.001	0.002	0.017	0.3
3.714 Å	0.003	0.009	-0.003	0.001	-0.001	0.002	0.017	0.3

Table S15. Parameters of electrochemical experiments performed by cyclic voltammetry at different sweep rates for the first reduction process for complex **1**.

Sweep rate	E_c, V	E_a, V	$E_{1/2}$, V	ΔE, mV	I_c*10^6, A	I_a*10^6, A
50 mV/s	-0.32	-0.21	-0.27	90	-2.9	1.6
100 mV/s	-0.32	-0.21	-0.27	90	-3.9	2.7
150 mV/s	-0.32	-0.21	-0.27	90	-4.8	3.6
200 mV/s	-0.32	-0.21	-0.27	90	-5.5	4.1

Table S16. Parameters of electrochemical experiments performed by cyclic voltammetry at different sweep rates for the oxidation process for complex **1**.

Sweep rate	E_c, V	E_a, V	$E_{1/2}$, V	ΔE, mV	I_c*10^6, A	I_a*10^6, A
50 mV/s	0.57	0.67	0.62	100	-1.1	2.4
100 mV/s	0.57	0.67	0.62	100	-2.0	3.9
150 mV/s	0.57	0.67	0.62	100	-2.8	5.3
200 mV/s	0.57	0.67	0.62	100	-3.4	6.0

Table S17. Parameters of electrochemical experiments performed by cyclic voltammetry at different sweep rates for the first reduction process for complex **3**.

Sweep rate	E_c, V	E_a, V	$E_{1/2}$, V	ΔE, mV	I_c*10^6, A	I_a*10^6, A
50 mV/s	-0.23	-0.15	-0.19	80	5.3	2.8
100 mV/s	-0.24	-0.14	-0.19	100	7.5	3.9
150 mV/s	-0.24	-0.14	-0.19	100	9.2	5.5
200 mV/s	-0.25	-0.14	-0.19	110	10.7	6.5

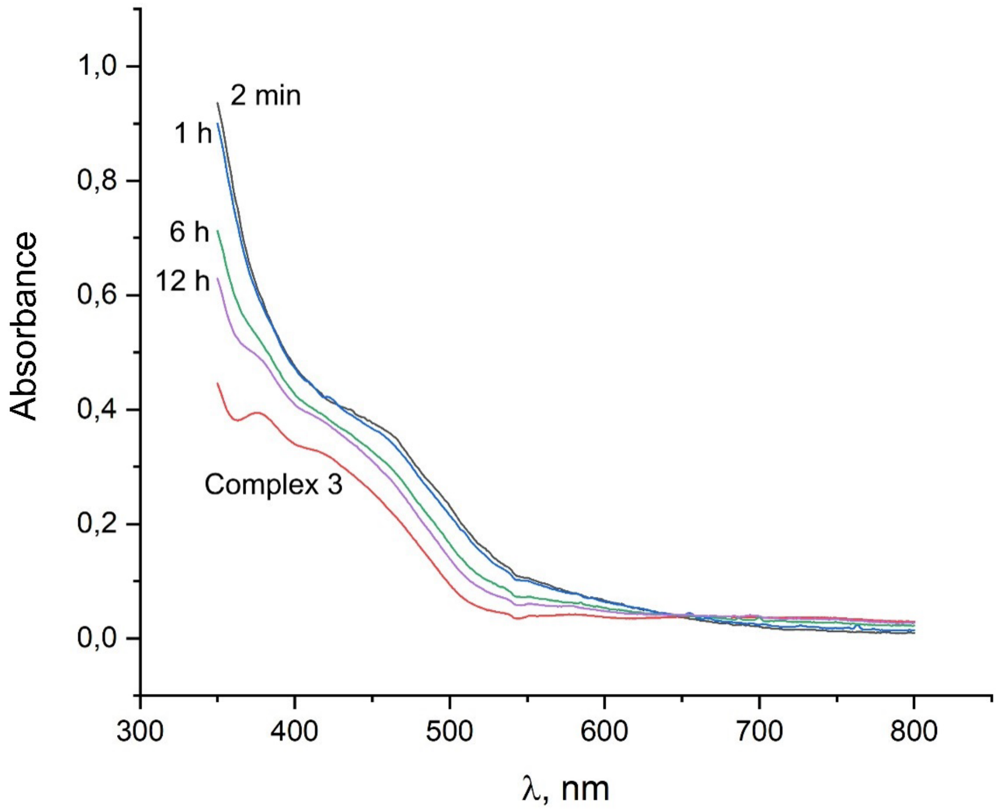

Figure S1. UV-vis spectra of **2** in CH_2Cl_2 depending on time. Red line is the UV spectrum of **3** under the same conditions.

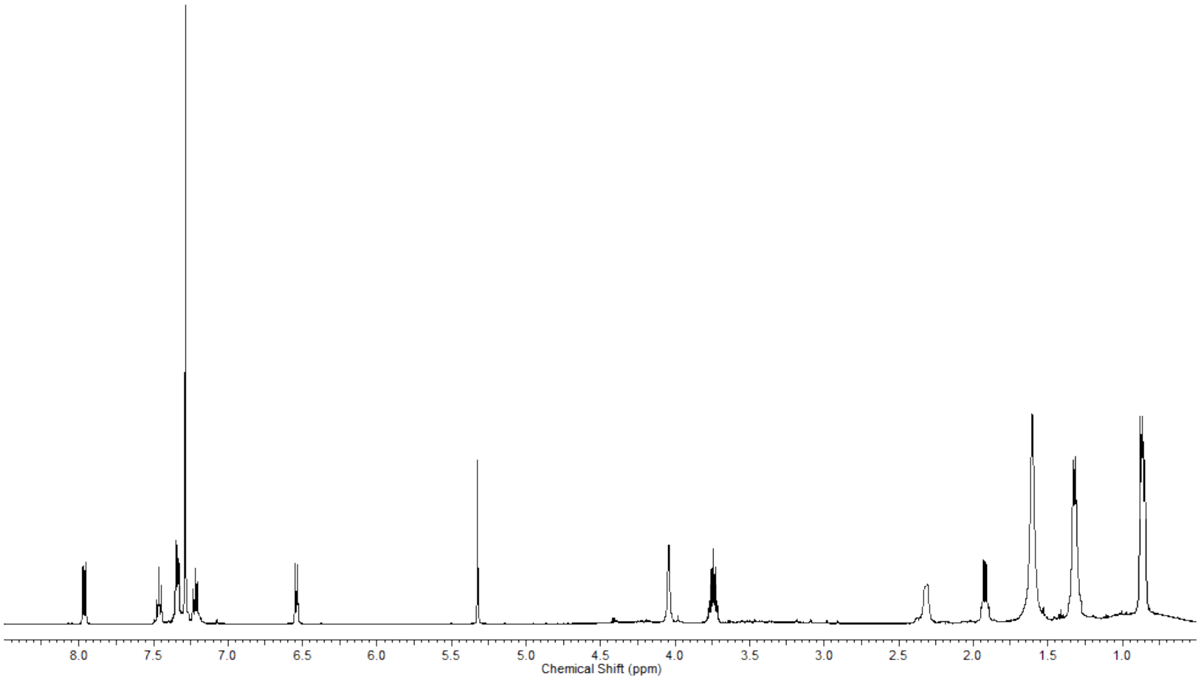

Figure S2. ^{1}H NMR spectrum of **1** in $CDCl_3$.

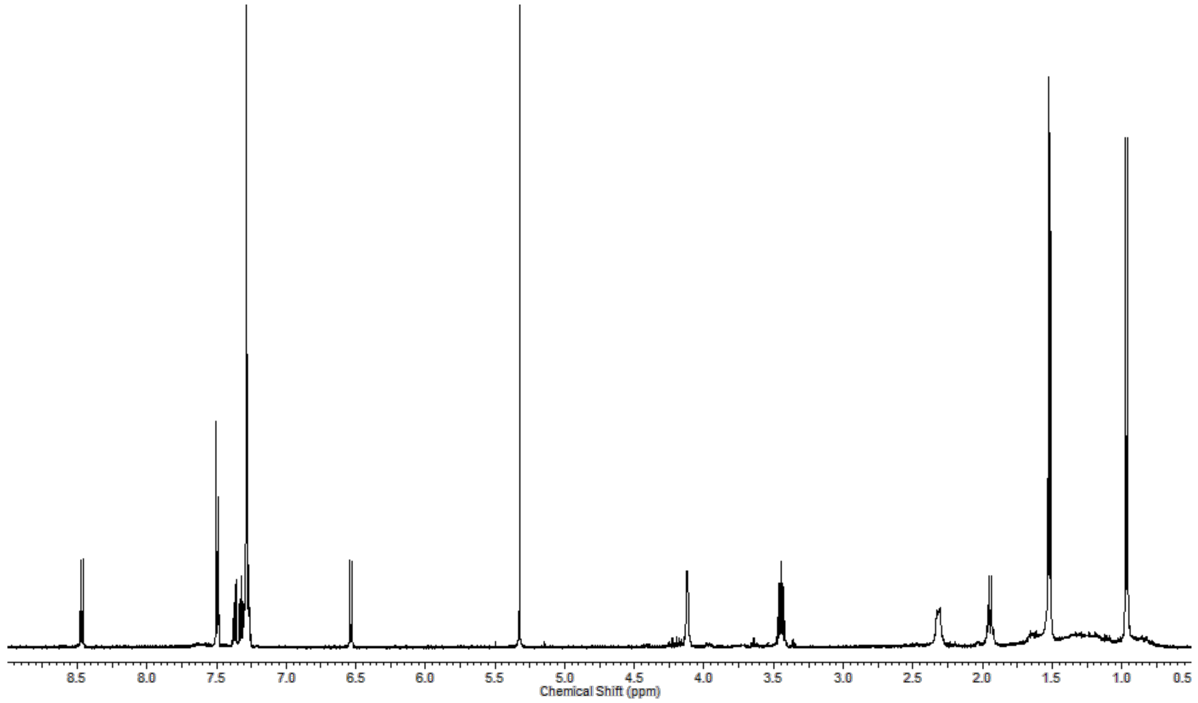

Figure S3. ^{1}H NMR spectrum of **3** in $CDCl_3$.

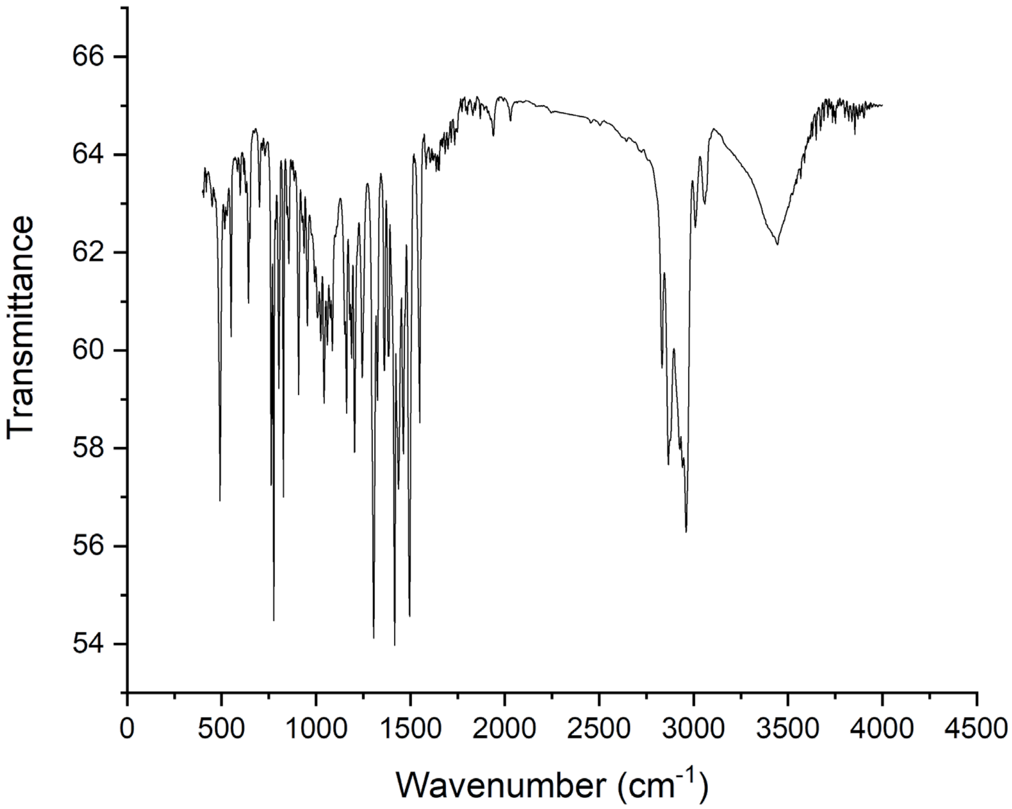

Figure S4. FT-IR spectrum of **1**.

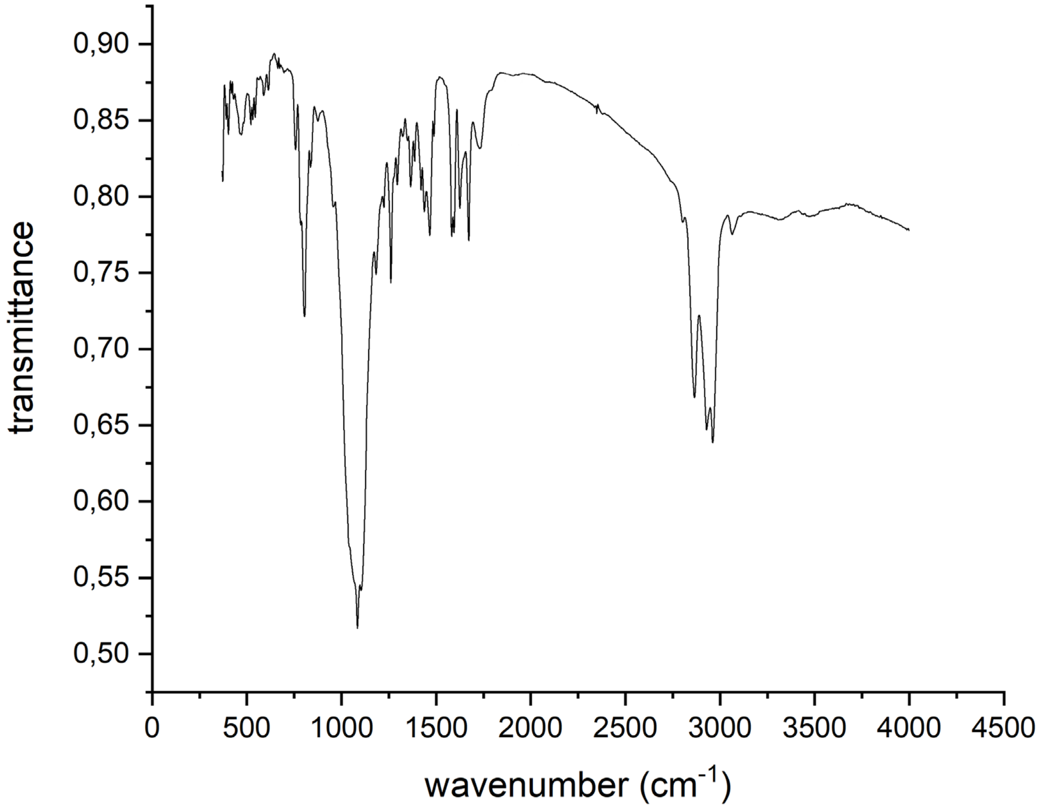

Figure S5. FT-IR spectrum of **2**.

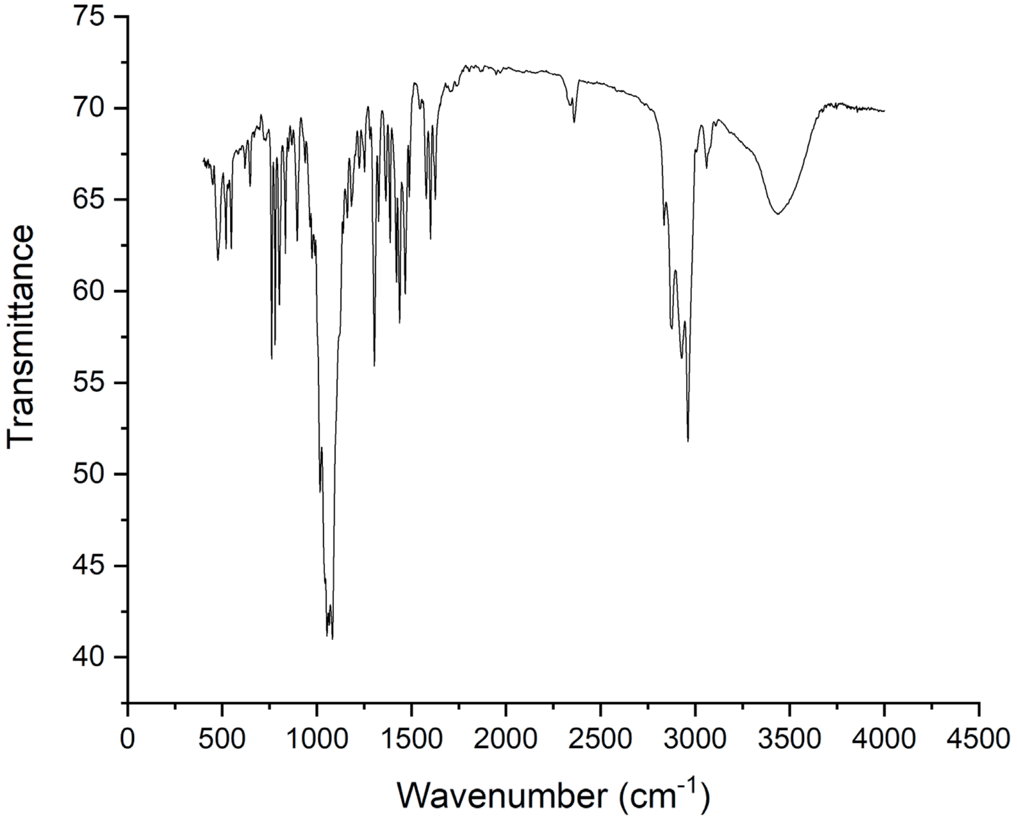

Figure S6. FT-IR spectrum of **3**.

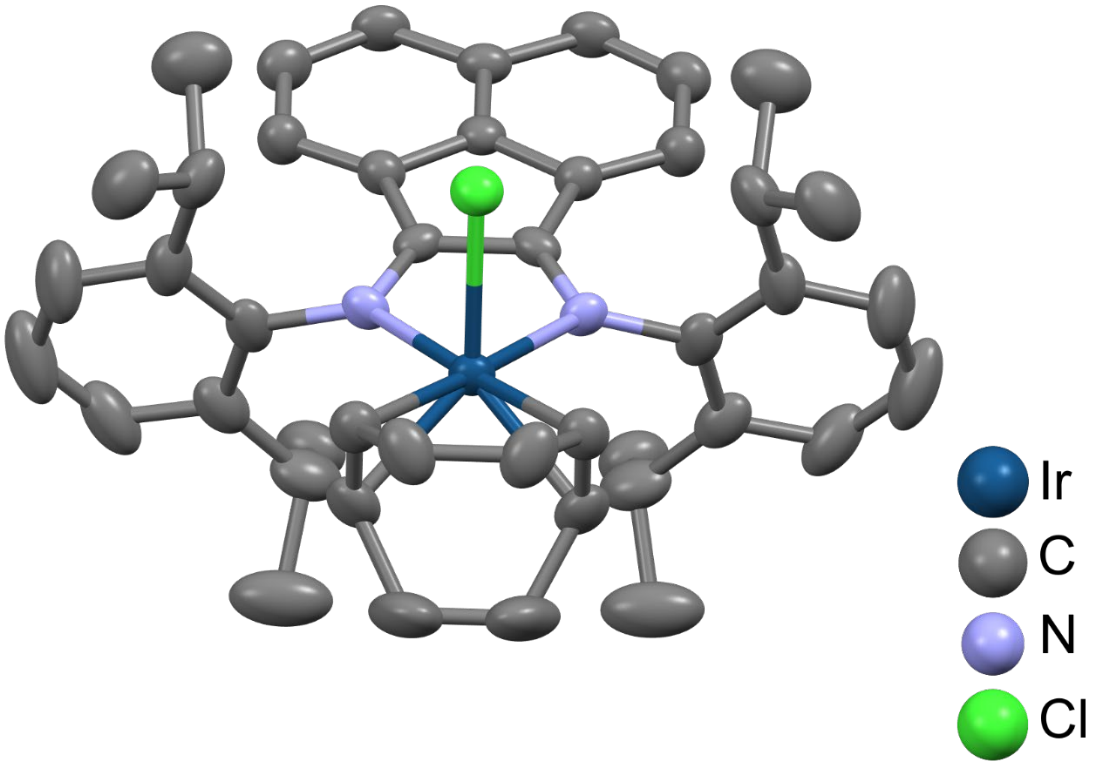

1

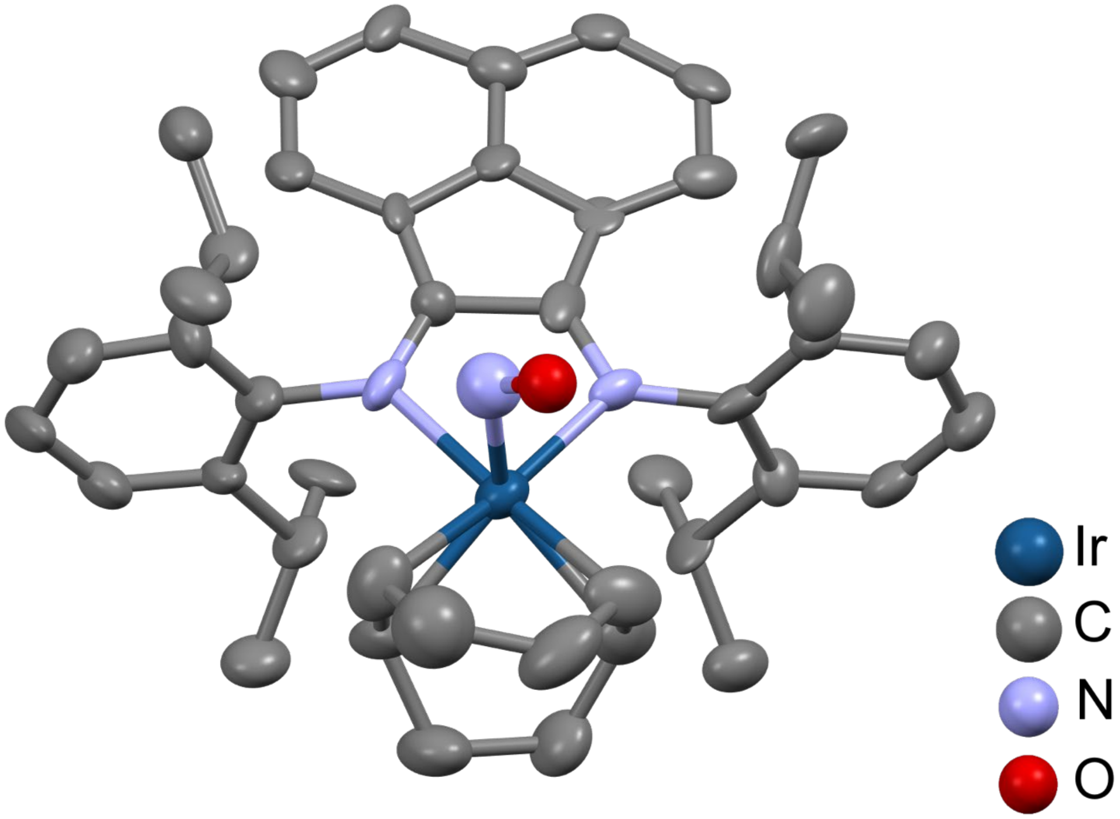

cation of **2**

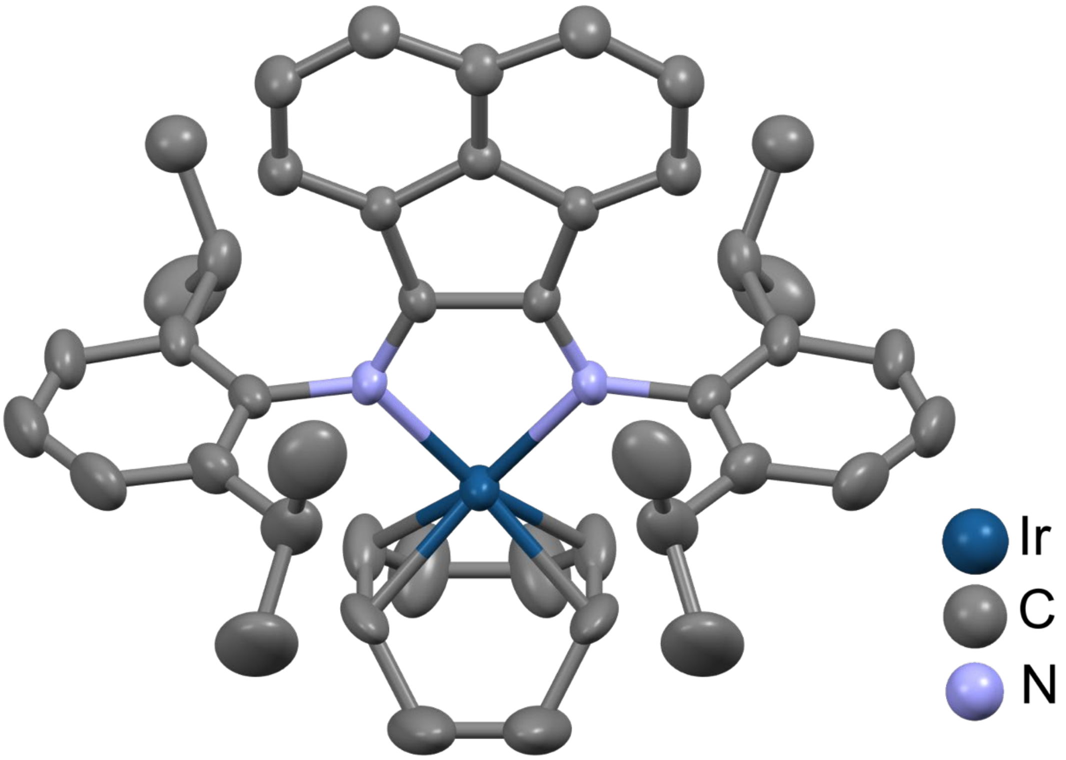

cation of **3**

Figure S7. ORTEP representation (50% probability ellipsoids) of **1**, cation of **2** and cation of **3**.

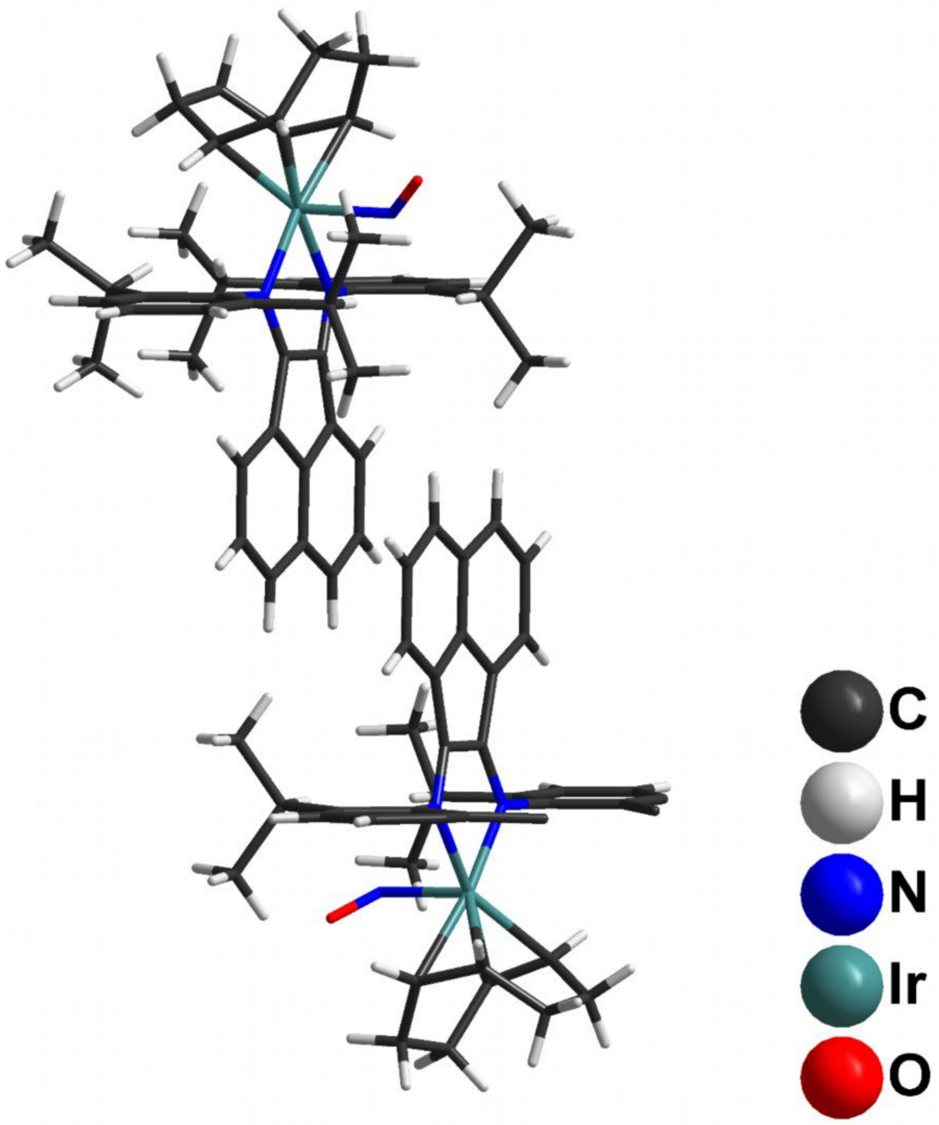

Figure S8. π-π connected dimers in the crystal packing of **2**.

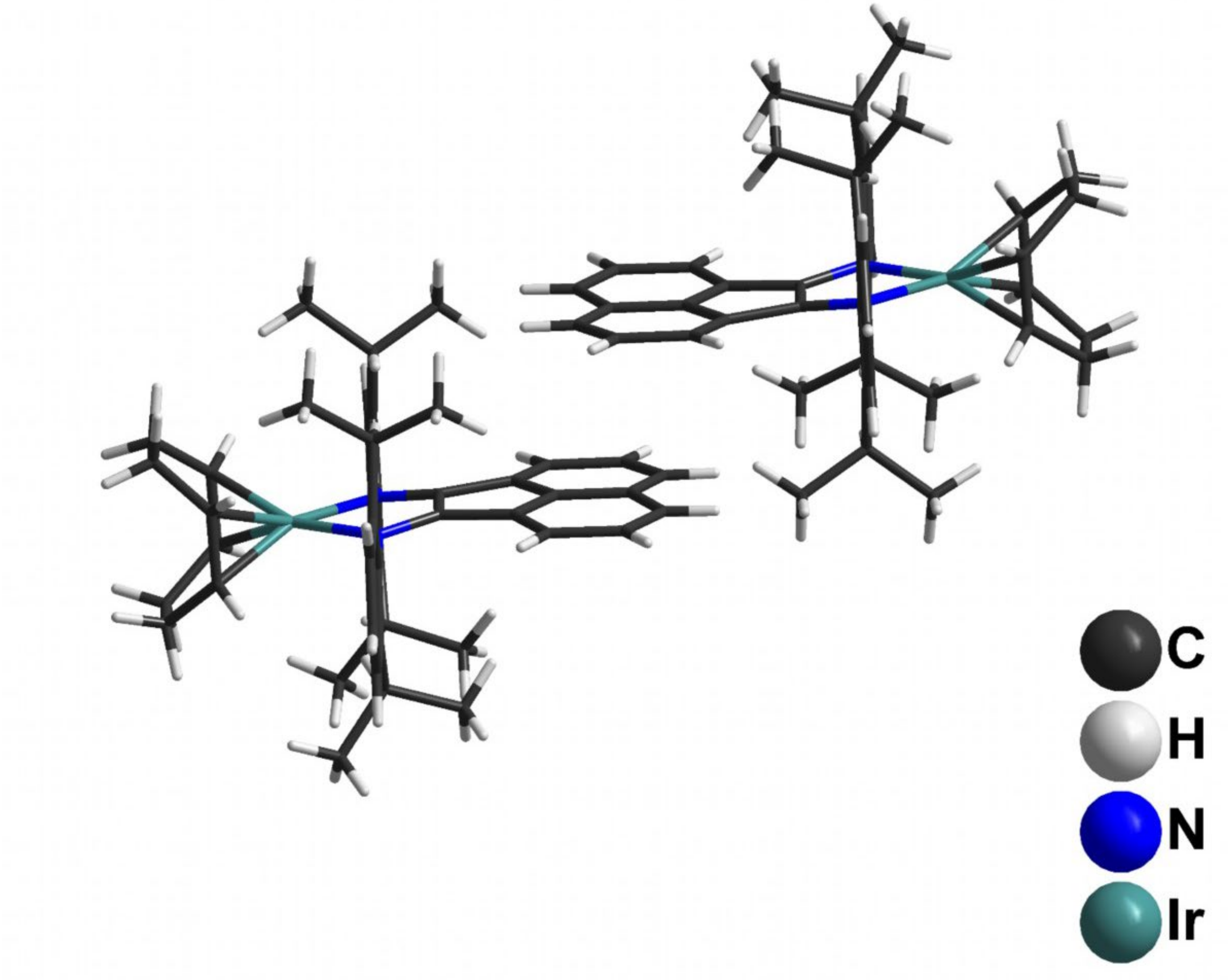

Figure S9. π-π connected dimers in the crystal packing of **3**.

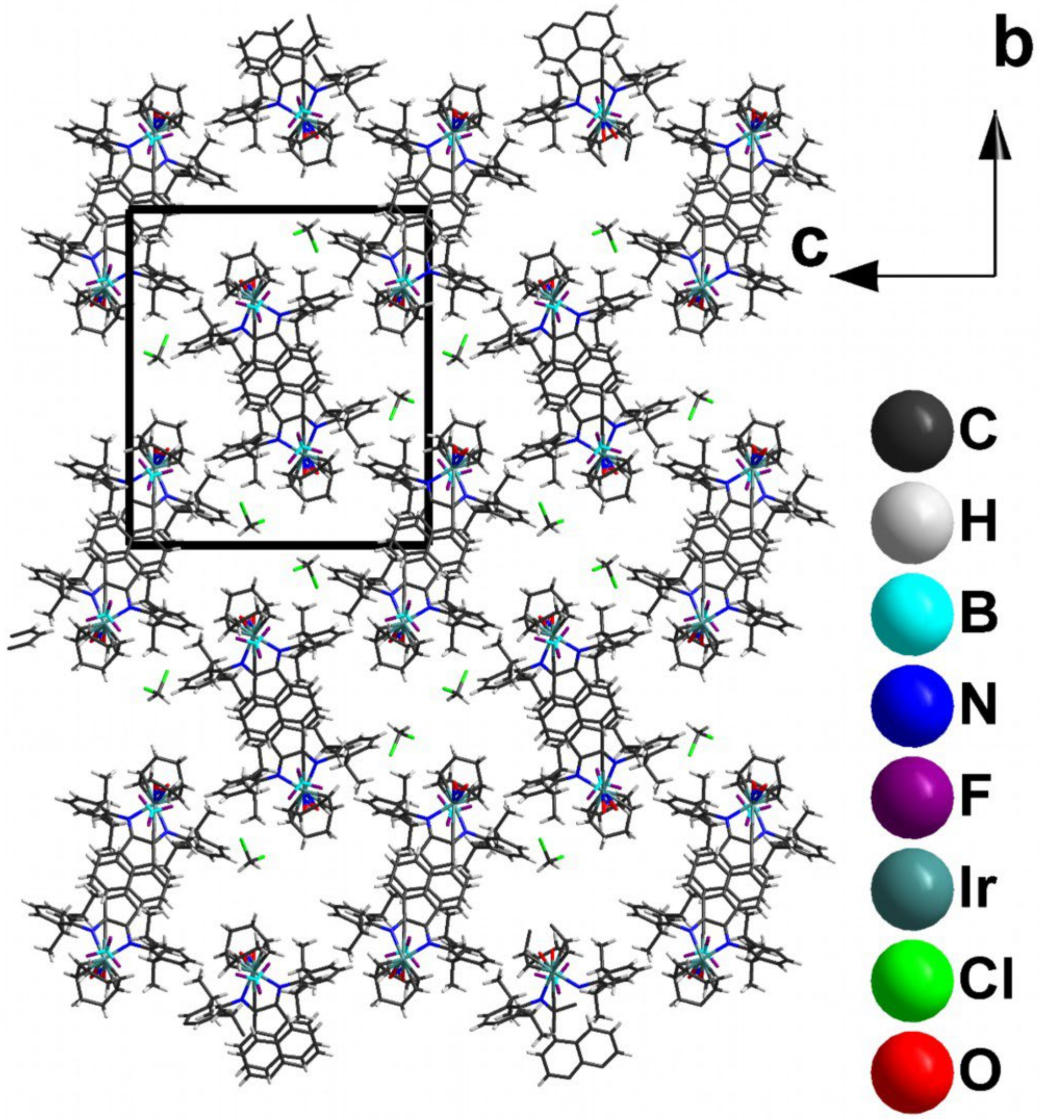

Figure S10. Crystal packing of **2**.

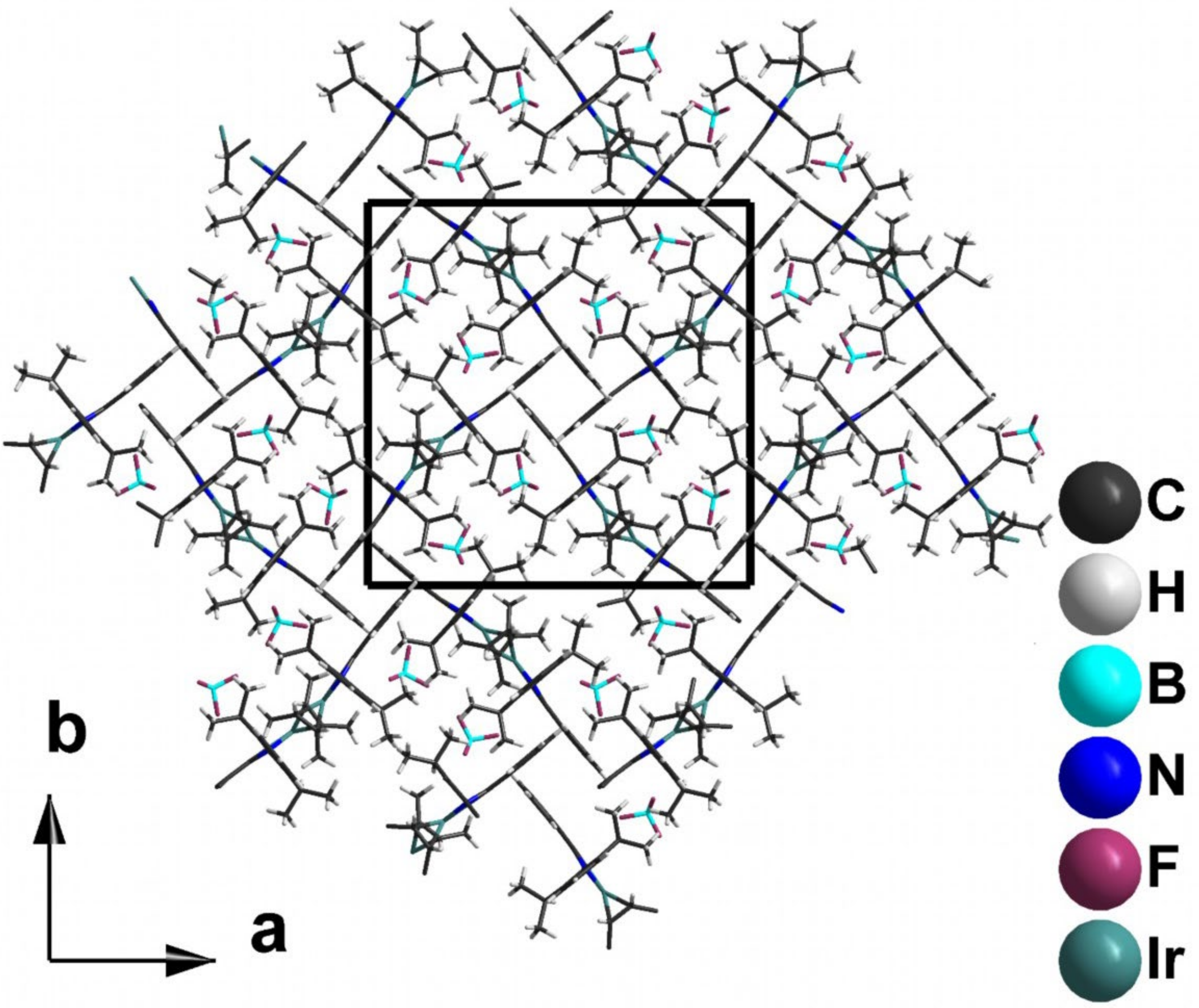

b
a
C
H
B
N
F
Ir

Figure S11. Crystal packing of **3**.

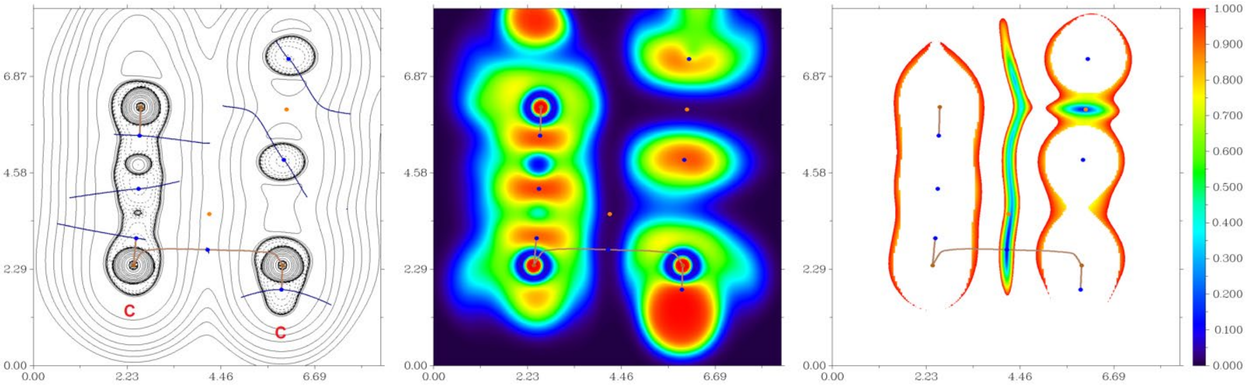

Figure S12. Contour line diagram of the Laplacian of electron density distribution, bond paths, and selected zero-flux surfaces (left panel), visualization of electron localization function (ELF, center panel) and reduced density gradient (RDG, right panel) analyses for selected intermolecular C···C contacts in the crystal structure **2**. Bond critical points are shown in blue, nuclear critical points – in pale brown, ring critical points – in orange, bond paths are shown as pale brown lines, length units – Å, and the color scale for the ELF and RDG maps is presented in atomic units.

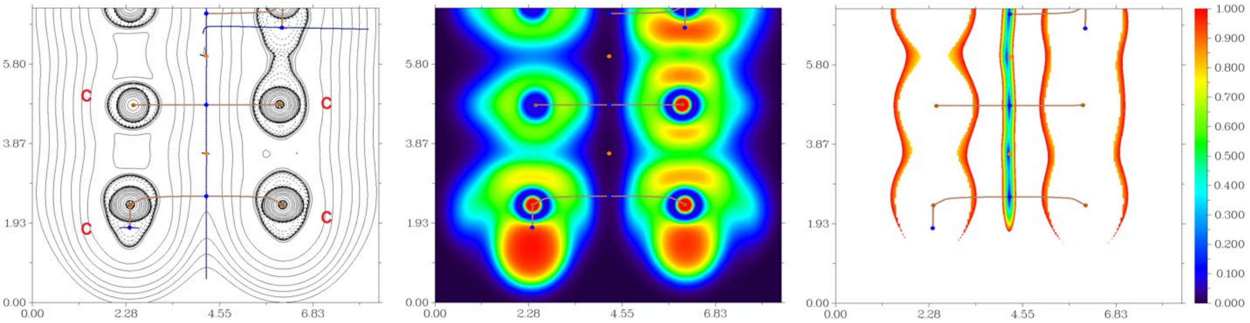

Figure S13. Contour line diagram of the Laplacian of electron density distribution, bond paths, and selected zero-flux surfaces (left panel), visualization of electron localization function (ELF, center panel) and reduced density gradient (RDG, right panel) analyses for selected intermolecular C···C contacts in the crystal structure **3**. Bond critical points are shown in blue, nuclear critical points – in pale brown, ring critical points – in orange, bond paths are shown as pale brown lines, length units – Å, and the color scale for the ELF and RDG maps is presented in atomic units.

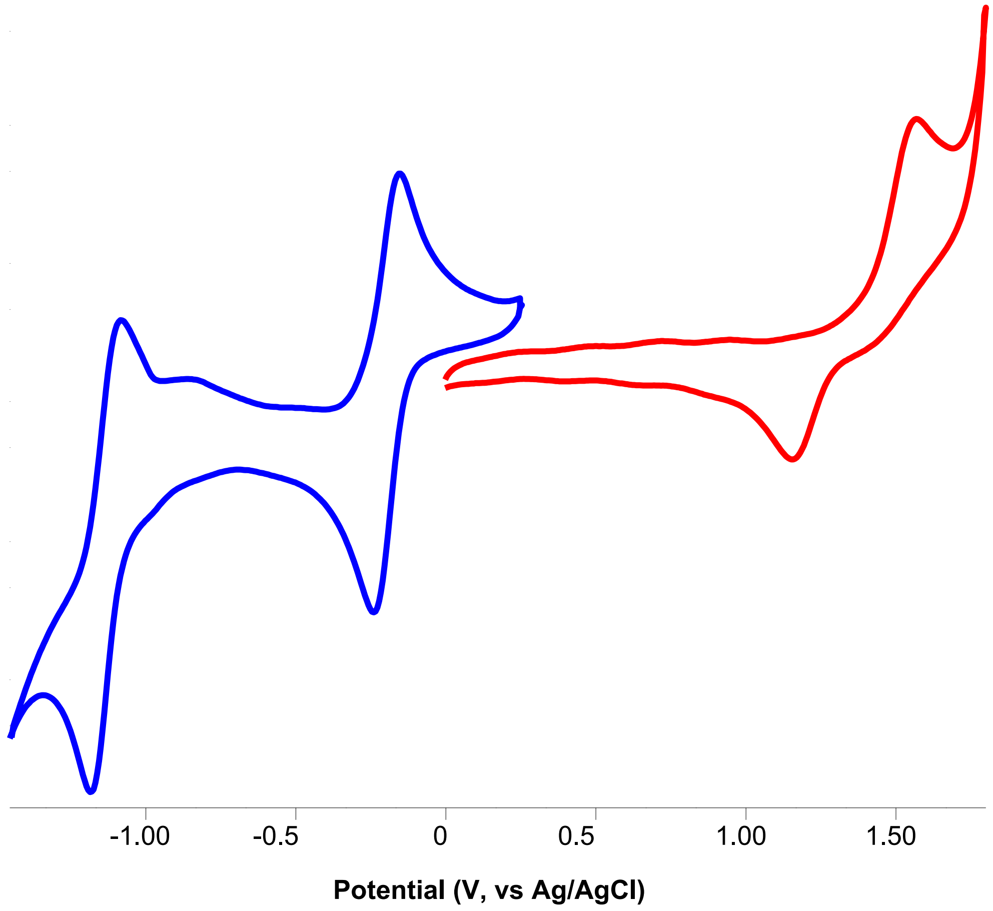

Figure S14. CV of **3** in CH_2Cl_2 in the -1.5–1.8 V region at potential scan rate of 100 mV/s (blue spectrum – reduction part, red spectrum – oxidation part).

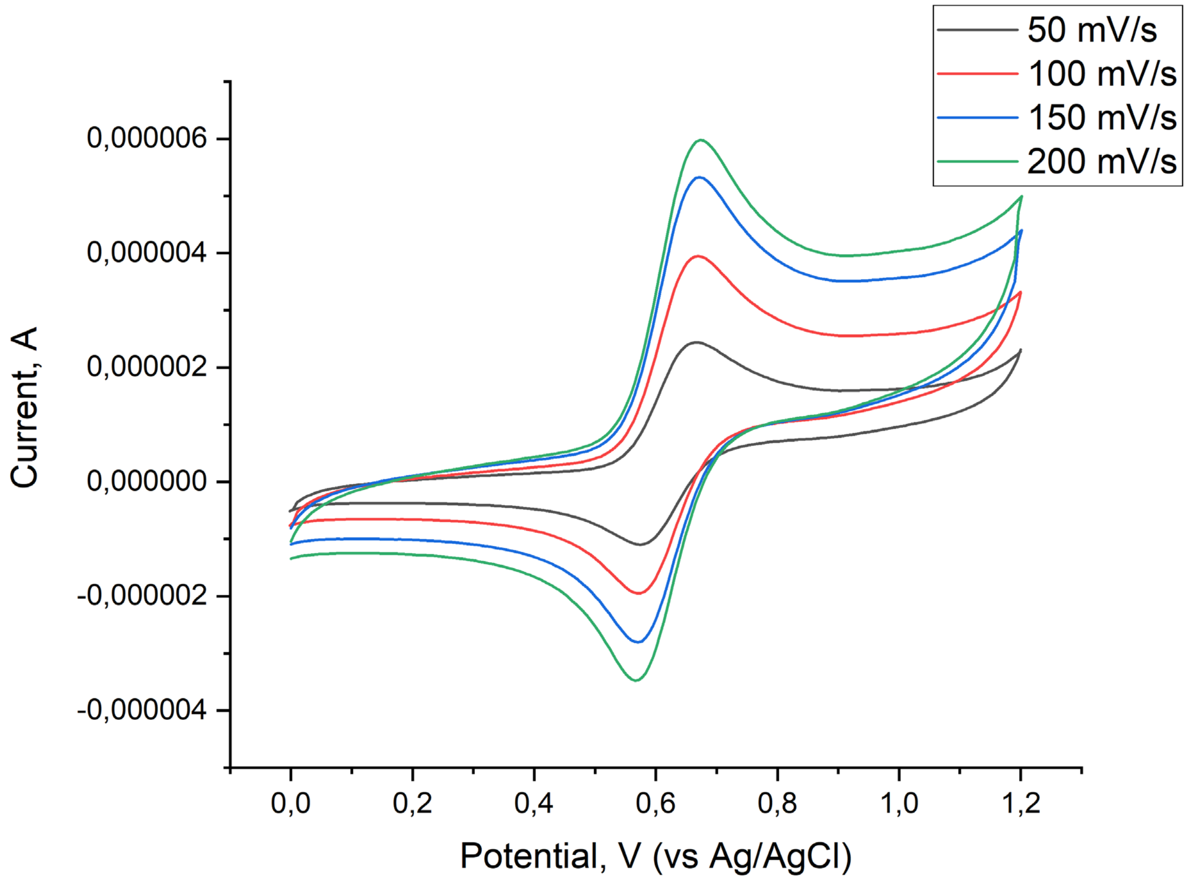

Figure S15. CVs of **1** in CH_2Cl_2 (oxidation process), recorded at various potential scan rates (50-200 mV/s).

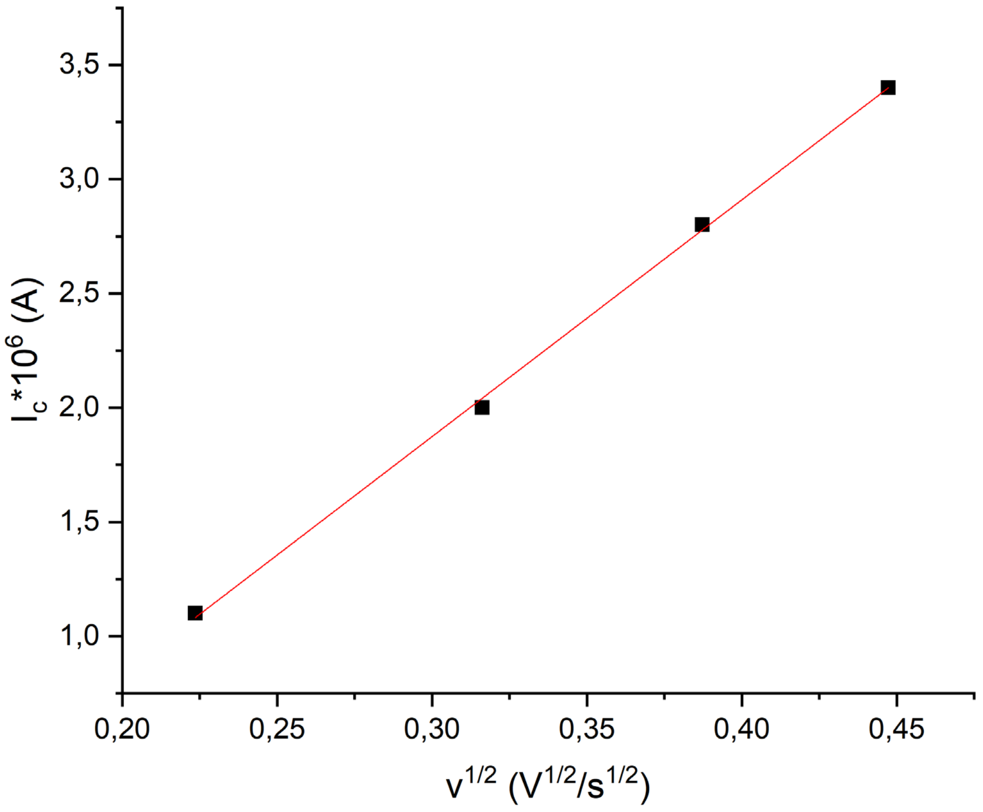

Figure S16. Dependence of the cathodic current on the square root of the sweep rate for the oxidation process for **1**.

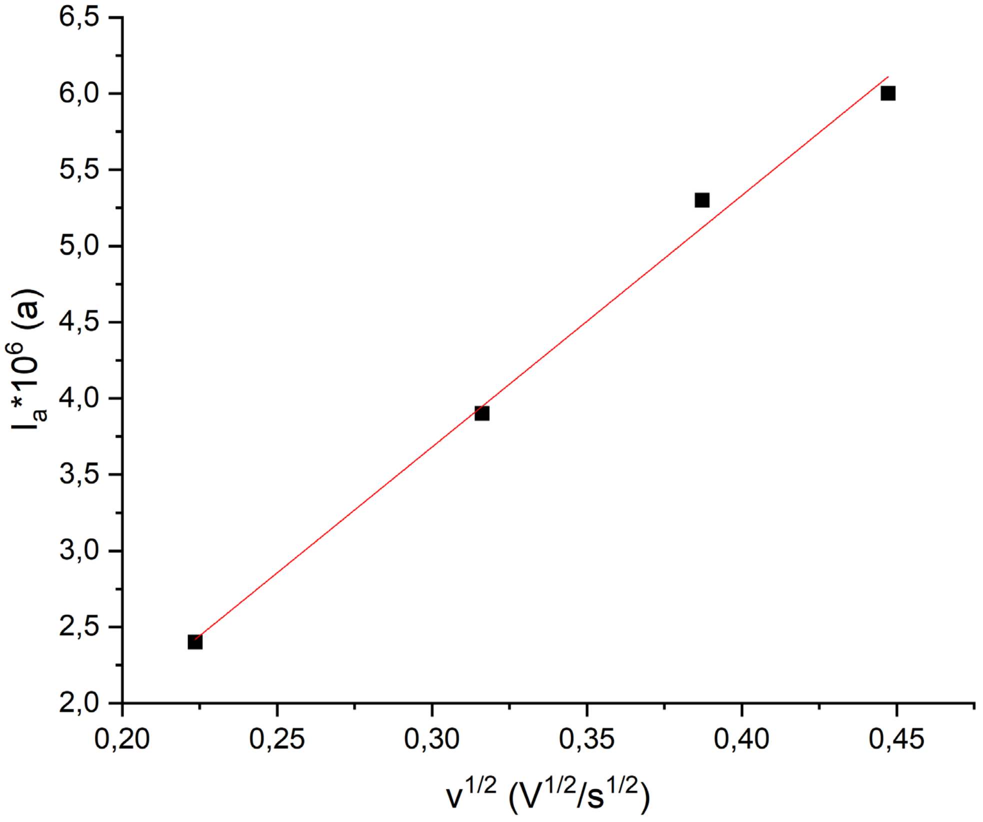

Figure S17. Dependence of the anodic current on the square root of the sweep rate for the oxidation process for **1**.

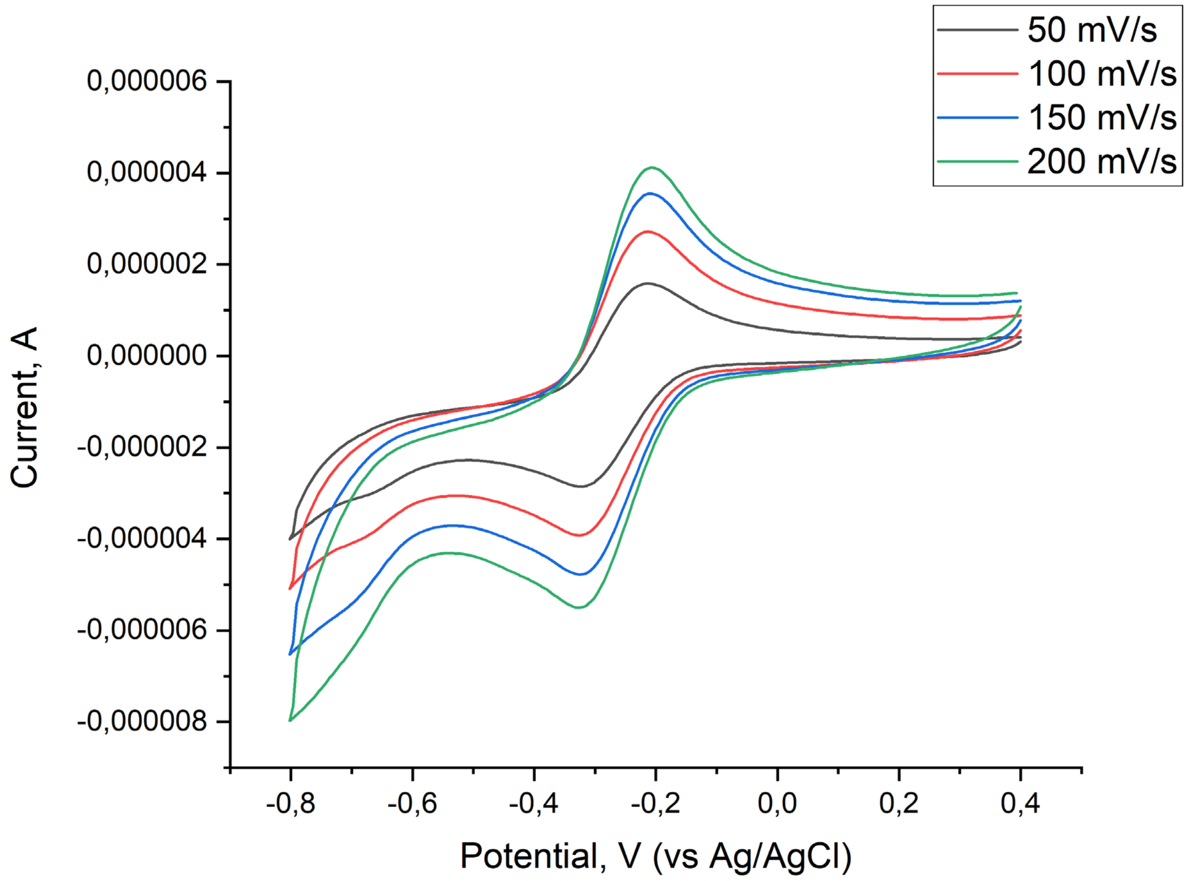

Figure S18. CVs of **1** in CH_2Cl_2 (first reduction process), recorded at various potential scan rates (50-200 mV/s).

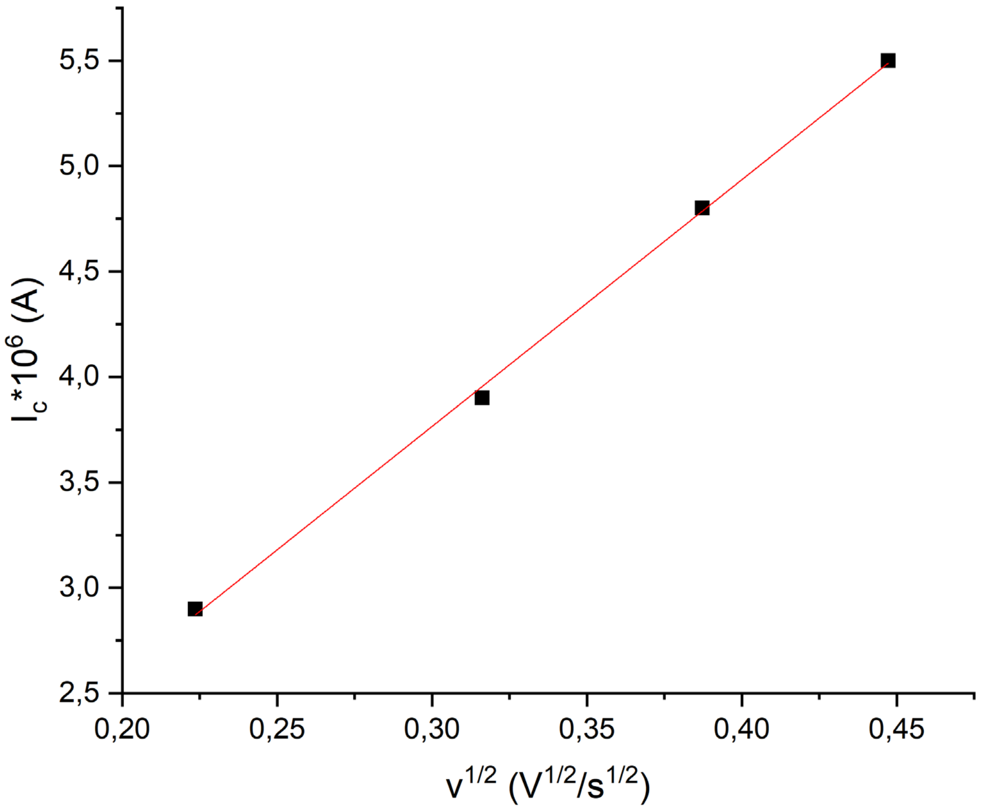

Figure S19. Dependence of the cathodic current on the square root of the sweep rate for the first reduction process for **1**.

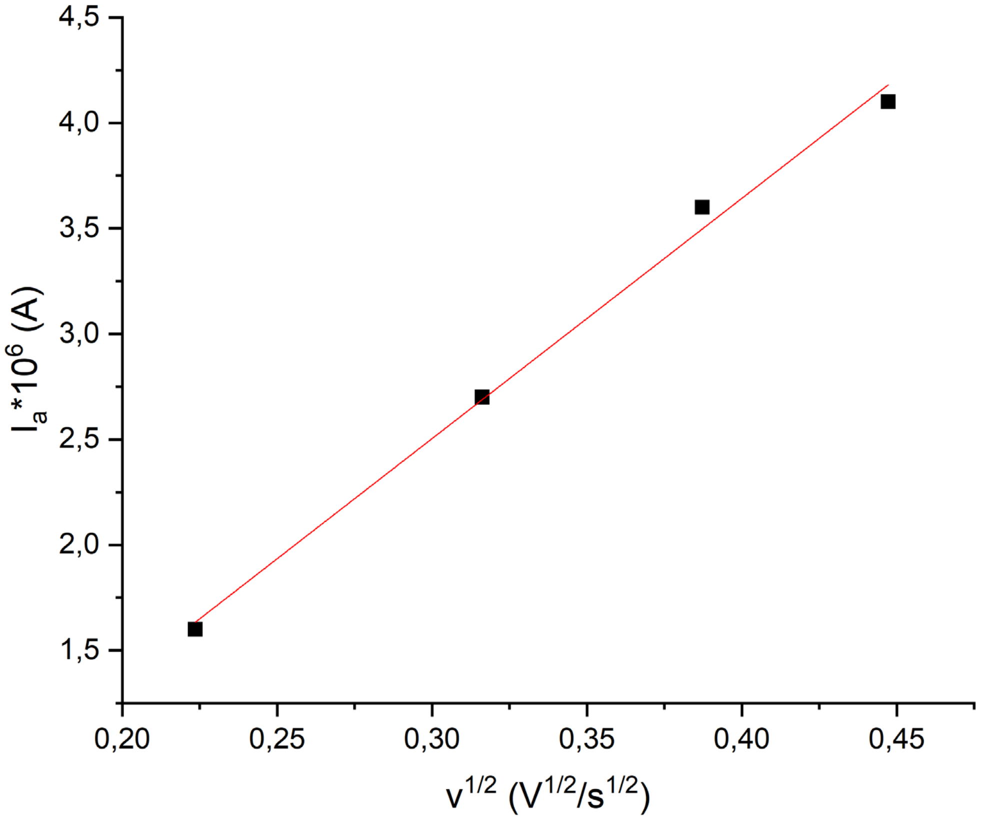

Figure S20. Dependence of the anodic current on the square root of the sweep rate for the first reduction process for **1**.

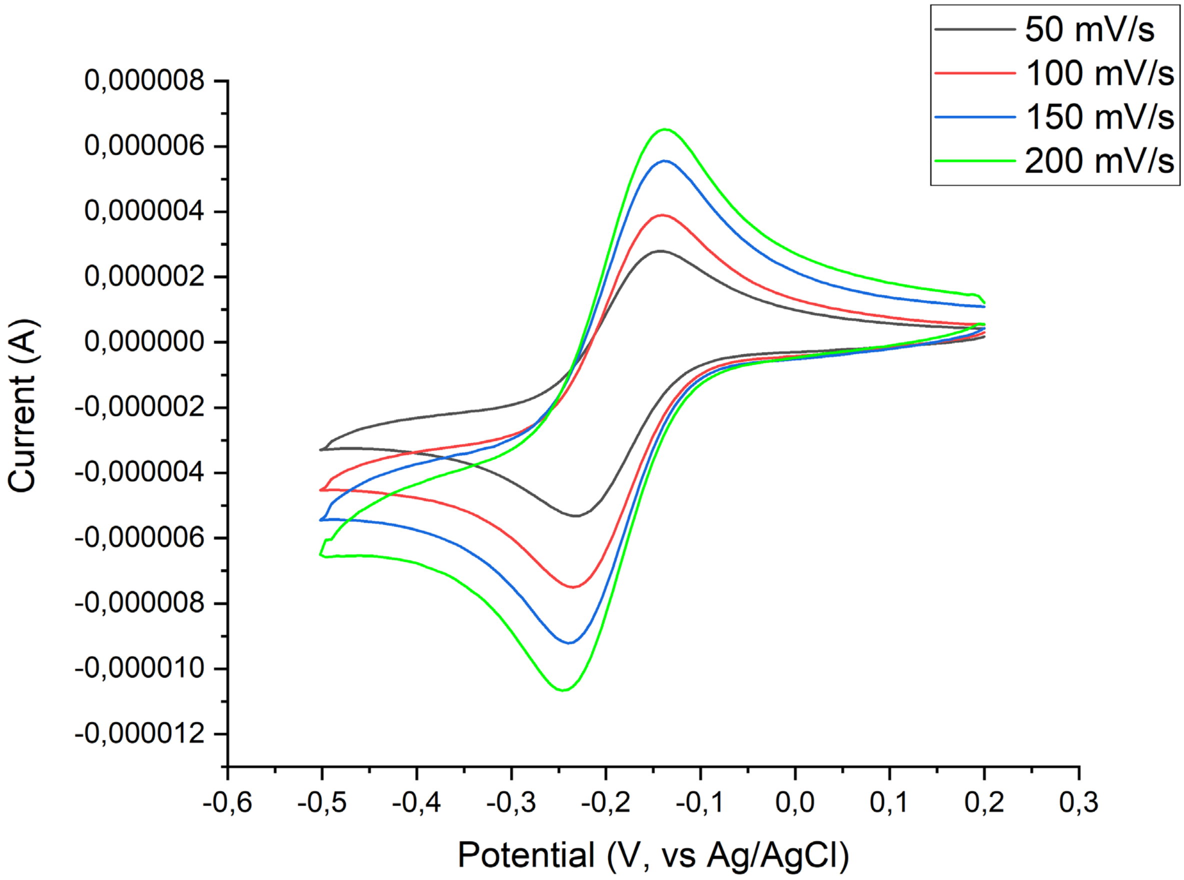

Figure S21. CVs of **3** in CH_2Cl_2 (first reduction process), recorded at various potential scan rates (50-200 mV/s).

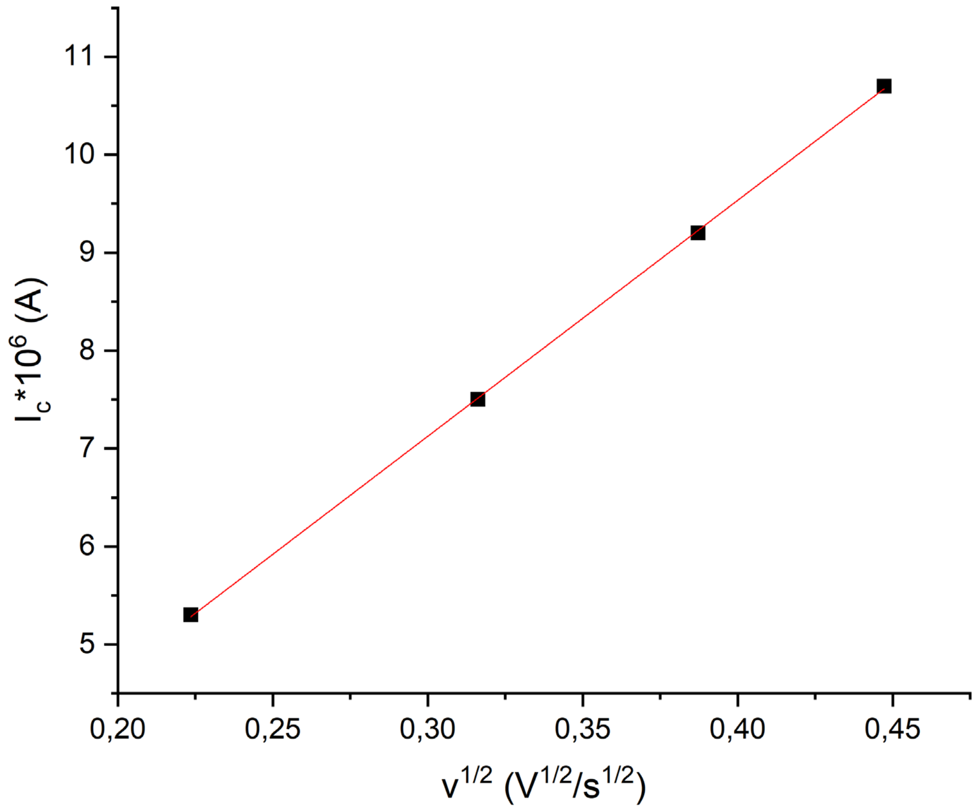

Figure S22. Dependence of the cathodic current on the square root of the sweep rate for the first reduction process for **3**.

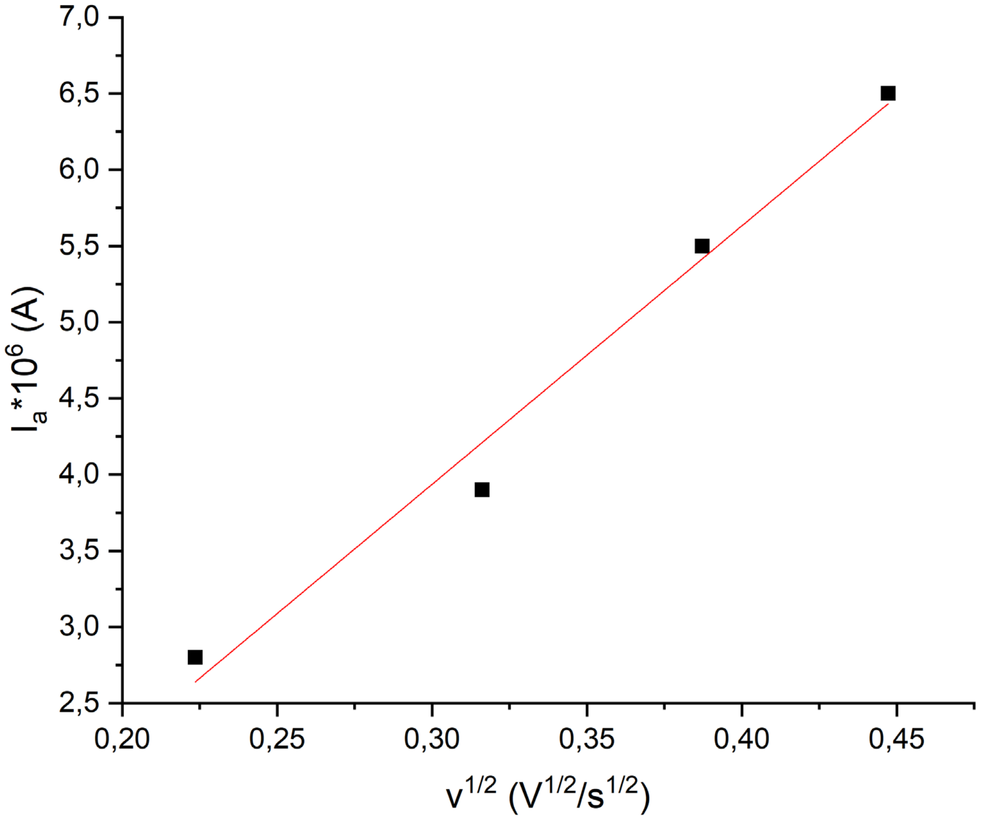

Figure S23. Dependence of the anodic current on the square root of the sweep rate for the first reduction process for **3**.

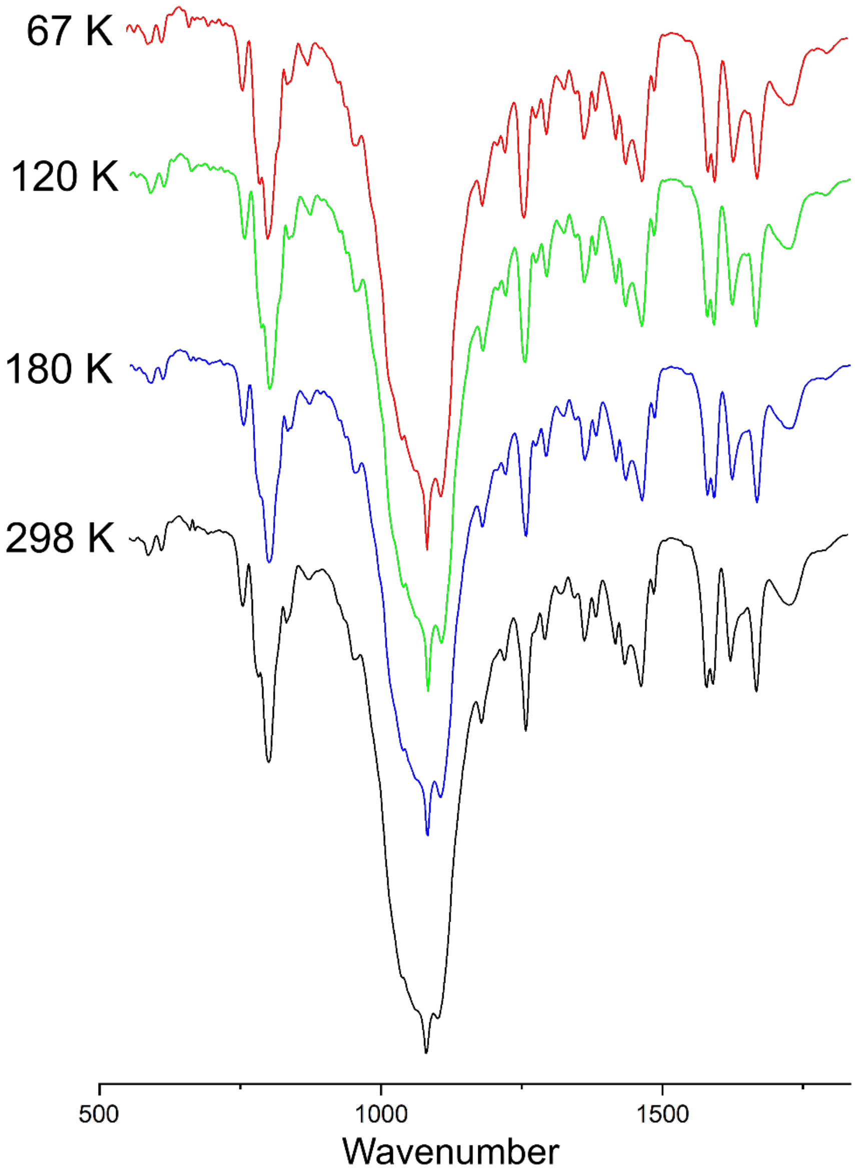

Figure S24. Dependence of the FT-IR spectrum of **2** on temperature.

FT-IR spectra of **2** show a broad band of vibrations of the nitroso group at 1721 cm^{-1}, as well as C=N vibration bands of dpp-bian in the region of 1672-1575 cm^{-1}. As the temperature decreases from 298 K to 69 K, no significant changes are observed: both the position and the intensity of all bands are preserved.

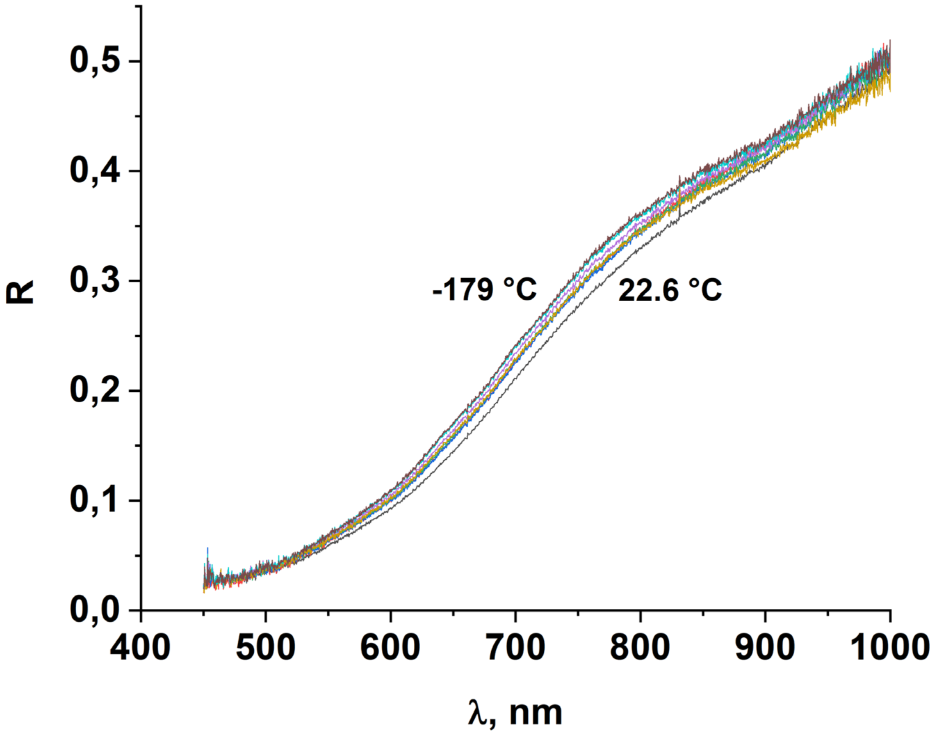

Figure S25. Dependence of the diffuse reflectance spectrum of **2** on temperature.

The diffuse reflectance spectra show strong absorption in the 450-550 nm region. Significant spectral changes depending on temperature are not observed. When the temperature is lowered to 67 K, a slight shift of absorption to the region of higher energies is observed, which is typical for any coordination compounds.

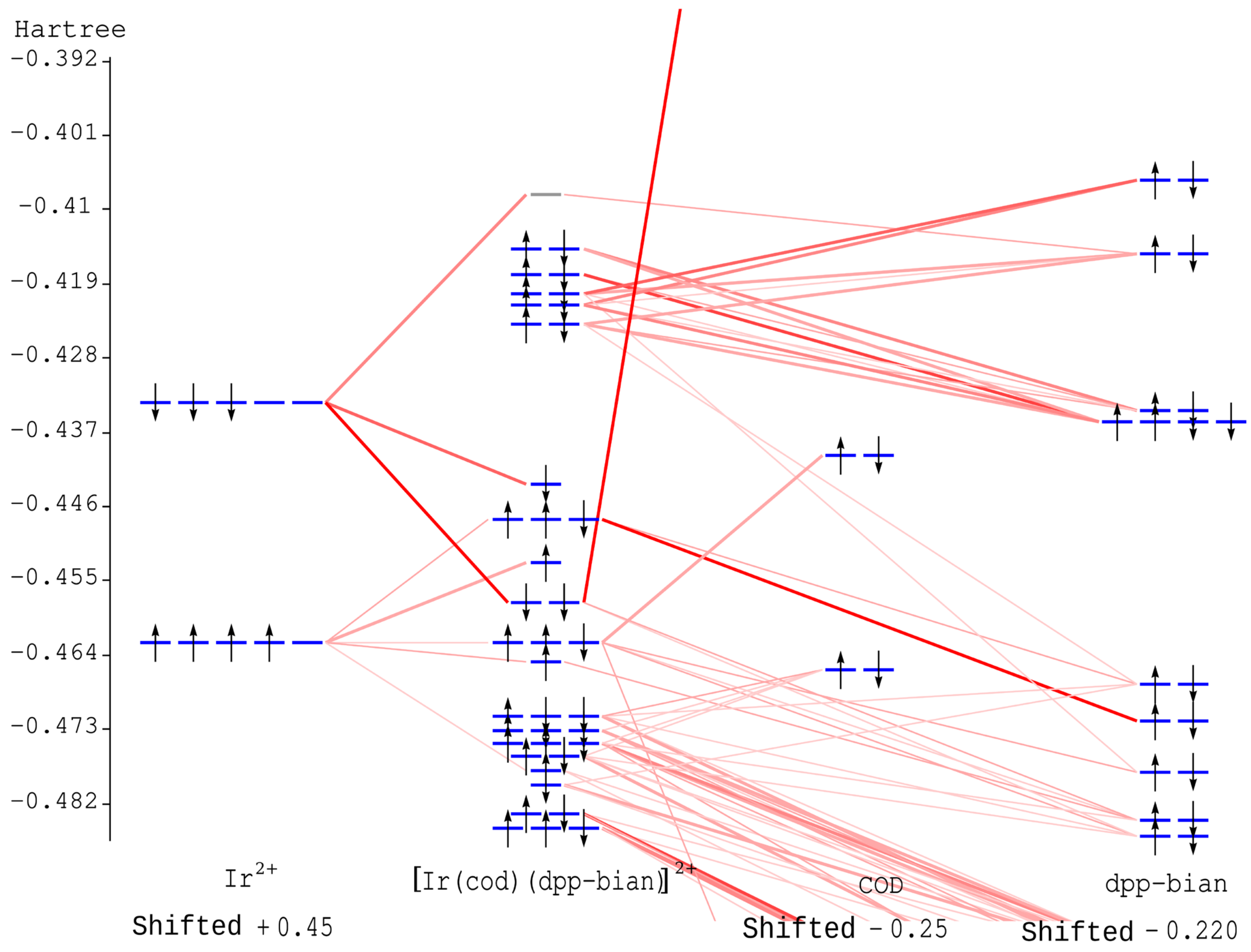

Figure S26. Electronic energy levels for $[Ir(cod)(dpp\text{-}bian)]^{2+}$ (cation of **4**) and its fragments.

Supplementary References

1. Romashev, N.F.; Gushchin, A.L.; Fomenko, I.S.; Abramov, P.A.; Mirzaeva, I. V.; Kompan'kov, N.B.; Kal'nyi, D.B.; Sokolov, M.N. A New Organometallic Rhodium(I) Complex with Dpp-Bian Ligand: Synthesis, Structure and Redox Behaviour. *Polyhedron* **2019**, *173*, 114110, doi:10.1016/j.poly.2019.114110.

2. Ayers, P.W.; Jenkins, S. Bond Metallicity Measures. *Comput. Theor. Chem.* **2015**, *1053*, 112–122, doi:10.1016/j.comptc.2014.10.040.
